# Femtosecond laser produced periodic plasma in a colloidal crystal probed by XFEL radiation

**DOI:** 10.1038/s41598-020-67214-z

**Published:** 2020-07-01

**Authors:** Nastasia Mukharamova, Sergey Lazarev, Janne-Mieke Meijer, Oleg Yu. Gorobtsov, Andrej Singer, Matthieu Chollet, Michael Bussmann, Dmitry Dzhigaev, Yiping Feng, Marco Garten, Axel Huebl, Thomas Kluge, Ruslan P. Kurta, Vladimir Lipp, Robin Santra, Marcin Sikorski, Sanghoon Song, Garth Williams, Diling Zhu, Beata Ziaja-Motyka, Thomas E. Cowan, Andrei V. Petukhov, Ivan A. Vartanyants

**Affiliations:** 1 Deutsches Elektronen-Synchrotron DESY, Notkestraße 85, D-22607 Hamburg, Germany; 2 National Research Tomsk Polytechnic University (TPU), pr. Lenina 30, 634050 Tomsk, Russia; 3 Debye Institute for Nanomaterials Science, University of Utrecht, Padualaan 8, 3508 TB Utrecht, The Netherlands; 4 University of California, 9500 Gilman Dr., La Jolla, San Diego, CA 92093 USA; 5 SLAC National Accelerator Laboratory, 2575 Sand Hill Rd, Menlo Park, CA 94025 USA; 6 Institute of Radiation Physics, Helmholtz-Zentrum Dresden-Rossendorf, 01328 Dresden, Germany; 7 Center for Advanced Systems Understanding (CASUS), Görlitz, Germany; 8 Technische Universität Dresden, 01069 Dresden, Germany; 9 Center for Free-Electron Laser Science, DESY, D-22607 Hamburg, Germany; 10 Department of Physics, Universität Hamburg, 20355 Hamburg, Germany; 11 Institute of Nuclear Physics, PAS, Radzikowskiego 152, 31-342 Krakow, Poland; 12 Laboratory of Physical Chemistry, Department of Chemical Engineering and Chemistry, Eindhoven University of Technology P.O. Box 513, 5600 MB Eindhoven, Netherlands; 13 National Research Nuclear University MEPhI (Moscow Engineering Physics Institute), Kashirskoe shosse 31, 115409 Moscow, Russia; 14 Present Address: Universiteit van Amsterdam, Science Park 904, 1090 GL Amsterdam, The Netherlands; 15 Present Address: Cornell University, Ithaca, NY 14850 USA; 16 Present Address: Division of Synchrotron Radiation Research, Department of Physics, Lund University, S-22100 Lund, Sweden; 17 Present Address: Lawrence Berkeley National Laboratory, 1 Cyclotron Rd, Berkeley, CA 94720 USA; 18 Present Address: European XFEL, Holzkoppel 4, D-22869 Schenefeld, Germany; 19 Present Address: NSLS-II, Brookhaven National Laboratory, Upton, NY, 11973-5000 USA

**Keywords:** Materials science, Optics and photonics, Physics

## Abstract

With the rapid development of short-pulse intense laser sources, studies of matter under extreme irradiation conditions enter further unexplored regimes. In addition, an application of X-ray Free-Electron Lasers (XFELs) delivering intense femtosecond X-ray pulses, allows to investigate sample evolution in IR pump - X-ray probe experiments with an unprecedented time resolution. Here we present a detailed study of the periodic plasma created from the colloidal crystal. Both experimental data and theory modeling show that the periodicity in the sample survives to a large extent the extreme excitation and shock wave propagation inside the colloidal crystal. This feature enables probing the excited crystal, using the powerful Bragg peak analysis, in contrast to the conventional studies of dense plasma created from bulk samples for which probing with Bragg diffraction technique is not possible. X-ray diffraction measurements of excited colloidal crystals may then lead towards a better understanding of matter phase transitions under extreme irradiation conditions.

## Introduction

Studies of materials at high-pressure conditions above Mbar are highly relevant to the physics of shock compressed matter^1–4^, planetary formation^5–7^, warm dense matter^2,4,8^, and different types of plasma-matter interactions^9,10^. The thermodynamic and transport properties of the high energy density material dictate its dynamics. The general understanding of processes in materials under high pressure and temperature such as phase transitions^2^ or phase separations^11^ are of a great scientific interest. Theoretical investigations of the dynamics of materials under high pressure are widespread, however, due to the limited diagnostic capabilities, experimental studies are still quite challenging.

There are currently two major methods of generating extreme high pressure, that are the static compression with diamond anvil cells and dynamic (shock wave) compression. The latter can be done, for example, by the powerful short-pulse lasers, which offer the possibility of creating ultra-high pressure, much higher than achievable in static compression experiments. The fundamental property of such high-power lasers is the creation of plasma at extreme pressure and temperature, which is causing a shock compression of the material. Dynamic shock compression of aluminum^8^, graphite^2^, and hydrocarbons^11^, as well as other materials driven by high-power lasers is a subject of recent extensive studies. The shock wave speed is in the range of several kilometers per second, therefore, a facility providing picosecond resolution is required for the *in situ* measurements of the shock-induced dynamics.

Newly developed X-ray Free-Electron Lasers (XFELs)^12–14^ are especially well suited for time-resolved measurements of the ultrafast structural dynamics of laser-created plasma^11,15^. XFELs provide extremely intense coherent femtosecond X-ray pulses, which are necessary to perform experiments with a time resolution that outperforms synchrotron sources by orders of magnitude^8,16^. X-ray scattering at an XFEL is a powerful tool for successful studies of the rapid changes in the material caused by a high-power infrared (IR) laser in both space and time^17^. Although the scattering signal from the uniform plasma is not very high, sufficient response can be achieved if the plasma is periodically modulated in space thus allowing the use of much stronger X-ray Bragg scattering^18^ and imaging^19–23^ techniques.

Such a unique form of matter as periodic plasma^24^ can be created, for example, by the high-power laser interaction with the periodically ordered dielectric material. Recently, the properties of periodic plasma have been studied theoretically^25,26^ and experimentally^27–30^. One of the fascinating properties of laser-produced periodic plasma is the enhancement of the generated intensity in the cases of low-order harmonic generation in comparison to a uniform plasma^31^. Therefore, investigation of dynamics and properties of a periodic plasma is beneficial for the development of laser-based radiation sources. In the present study a periodic plasma was formed by the IR laser interaction with colloidal crystals made of polystyrene.

Polystyrene, consisting of carbon and hydrogen, is an ideal model system for creating a plasma by the IR laser sources because these atoms have a relatively low ionisation threshold, accessible by high-power IR laser. This material is also of high relevance to the biological community, since most of the biological samples consist of light atoms such as carbon, nitrogen, oxygen, and hydrogen^32,33^. In addition, hydrocarbons are one of the most common chemical species throughout the Universe^34^. A considerable amount of hydrocarbons compressed to 150 GPa exists inside giant planets, especially icy giants such as Neptune and Uranus^35^. Also, some extrasolar planets^36^ and white dwarf stars^37^, are built from high-pressure carbon which was recently extensively studied^38^.

Here, we present *in situ* IR pump–X-ray probe diffraction experiment performed at an XFEL on the periodic polystyrene colloidal crystals. The periodicity of the sample allowed us to apply the Bragg peaks analysis and to observe dramatic ultrafast changes in the colloidal crystal sample. The experiment was performed with the IR laser intensity on the order of 10^14^ W/cm^2^. With such high intensities a confined hot periodic plasma was created and a shock wave was generated that compressed the surrounding pristine material. This shock wave reached pressures on the order of 100 GPa, triggering fast changes in the colloidal crystal structure. We found a good correspondence between the characteristic times determined in the experiment and in simulations. Below we discuss the details of the pump-probe experiment and theoretical modeling of the processes involved in our studies.

## Results

### Pump-probe experiment

The pump-probe experiment was performed at the Linac Coherent Light Source (LCLS)^12^ X-ray Pump Probe (XPP) beamline^39^ (see^40^ and Methods for experimental details). The colloidal crystal films were prepared from polystyrene spheres with a diameter of 163 ± 3 nm, using the vertical deposition method^41^. The grown colloidal crystal films consisted of 30–40 layers of close-packed hexagonal planes. The thickness of the film was slightly depending on the position on a film along the growth direction. The experiments were executed with three different IR laser intensities, I_1_ = 3.0.10^14^ W/cm^2^, I_2_ = 4.8.10^14^ W/cm^2^, and I_3_ = 6.3.10^14^ W/cm^2^ Table 1. For each IR laser intensity a pump-probe experiment was performed with a time delay variation *τ* from −10 ps to 1000 ps with the 25.25 ps time increment. Additionally, for two higher IR laser intensities (*I*_2_ and *I*_3_) we also accomplished measurements from −10 ps to 48.5 ps with the 6.5 ps time increment. Due to a sample degradation, measurements for each time delay were performed at a new position of the sample.

**Table 1:** Results of the experimental data analysis. IR laser parameters and the results of the Bragg peaks evaluation giving characteristic times for the integrated intensity and the size of the Bragg peaks in radial and azimuthal directions as a function of IR intensity (see Fig. 2).

Intensity, 10^14^ W/cm^2^	3.0	4.8	6.3
Laser fluence, J/cm^2^	16	25.5	33.6
Short times of intensity change, ps	—	3 ± 10	7.9 ± 1.1
Long times of intensity change, ps	299 ± 33	300 ± 28	275 ± 28
Times of radial peaks FWHM change, ps	302 ± 32	279 ± 50	425 ± 90
Times of azimuthal peaks FWHM change, ps	345 ± 235	353 ± 86	410 ± 78

**Figure 1:**
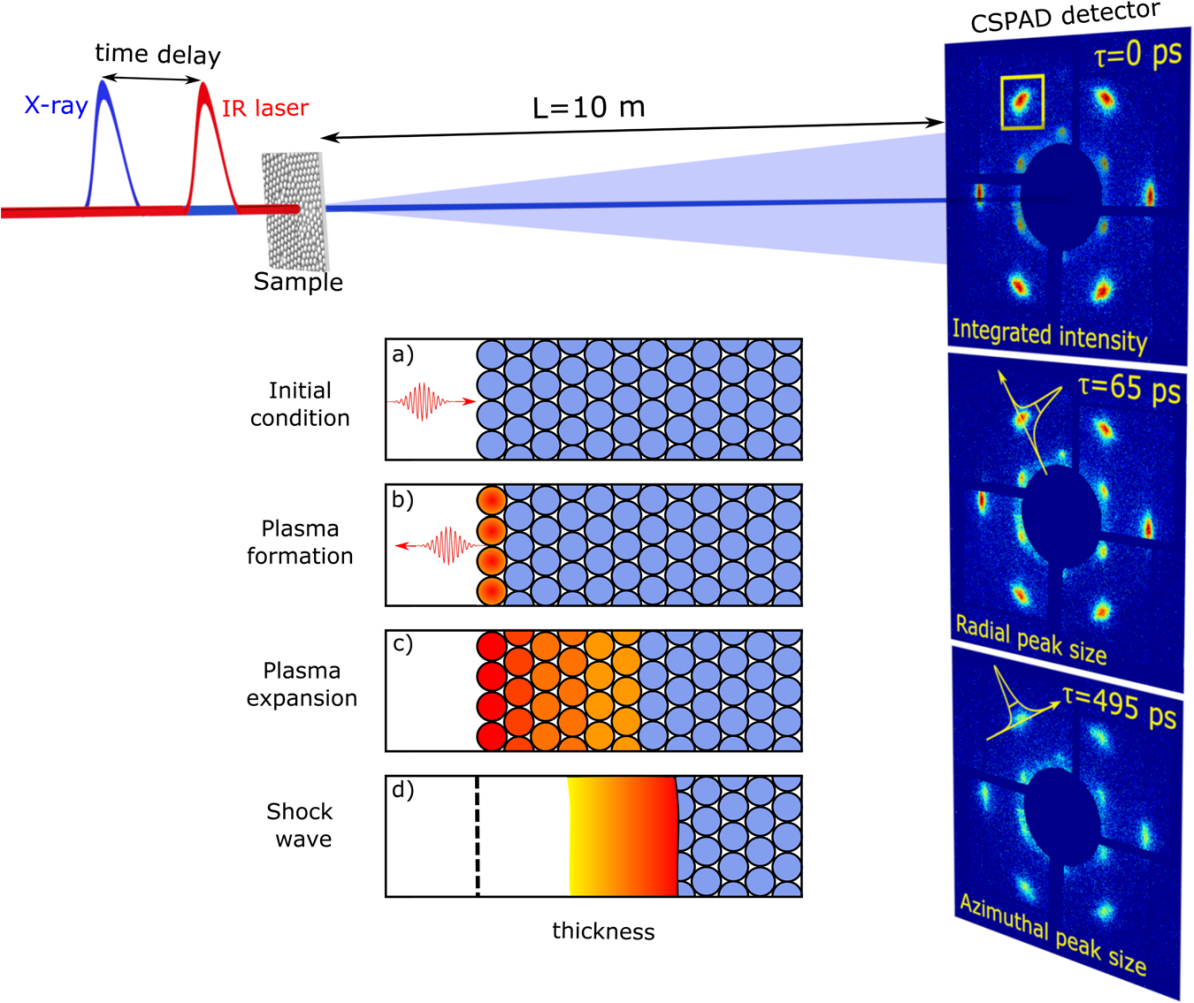
Scheme of the pump-probe experiment. XFEL pulses generated by the undulator are monochromatized by the diamond crystals and focused by the compound refractive lenses (not shown) to the size of 50 *μ*m at the sample position. CSPAD detector is positioned 10 m downstream from the colloidal sample. Evolution of diffraction patterns as a function of time delay between the IR pump laser and X-ray probe laser is shown on the right. Insets (**a**–**d**) visualize three-stage model of the IR laser-matter interaction. The colloidal particles are shown as circles. The color of the particles corresponds to the temperature of the colloidal crystal - red is plasma and blue is the cold material. The incoming IR laser pulse is pointing in the direction of the pulse propagation. Initially, the IR laser pulse is propagating towards the colloidal crystal sample and after interaction with the sample it is reflected by the created plasma on the top layer of the colloidal crystal. The top surface level of the initial colloidal crystal is marked by the black dashed line in (**d**).

**Figure 2:**
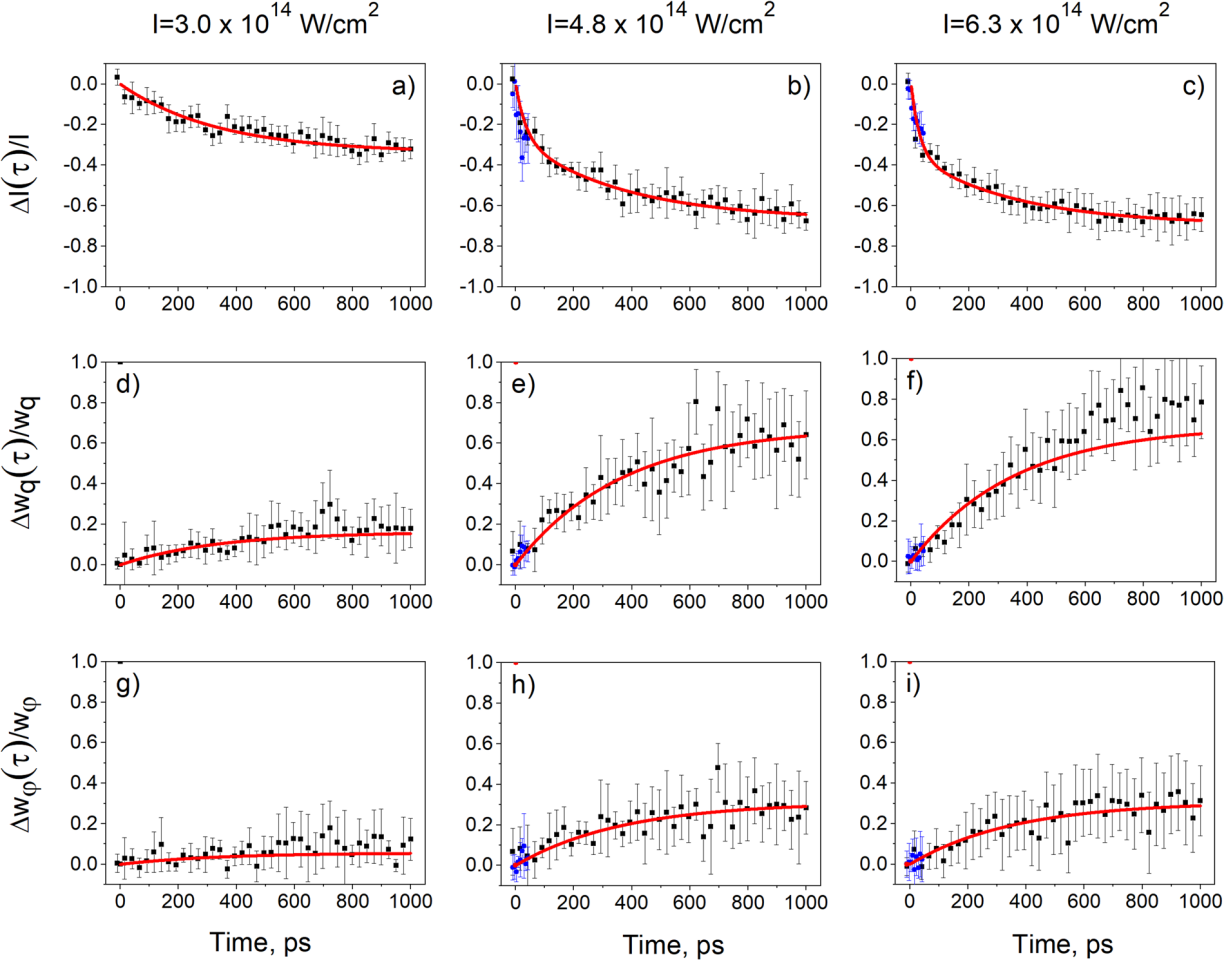
Time dependence of the relative change of the integrated intensity of the Bragg peaks $\Delta I(\tau )/I$ (**a**–**c**) and their widths in the radial $\Delta {w}_{q}(\tau )/{w}_{q}$ (**d**–**f**) and azimuthal $\Delta {w}_{\varphi }(\tau )/{w}_{\varphi }$ (**g**–**i**) directions at three measured IR laser intensities. Black (blue) dots are experimental data corresponding to 25.25 ps (6.5 ps) time delay increment and solid red lines are exponential fits.

Typical single-shot diffraction patterns for three different time delays are shown in the insets in Fig. 1. Bragg peak parameters corresponding to the most intense 110 reflections such as integrated intensity (*I*) and the peaks Full Width at Half Maximum (FWHM) in the radial (*w*_*q*_) and azimuthal (*w*_*φ*_) directions were analyzed. For three IR laser intensities variation of the Bragg peak intensity Δ*I*(*τ*)/*I*, radial $\Delta {w}_{q}(\tau )/{w}_{q}$, and azimuthal $\Delta {w}_{\phi }(\tau )/{w}_{\phi }$ broadening of the Bragg peaks are shown in Fig. 2 as a function of time delay (see Methods for the details of the diffraction data analysis).

The experimental measurements in Fig. 2 are depicted by black dots for 25.25 ps time increment and by blue dots for 6.5 ps time increment. The decay of the relative Bragg peak intensity accompanied by the growth of the peaks size (FWHM) is well visible for all three measured IR laser intensities. For two higher IR laser intensities, an additional fast drop of the relative intensity during the first picoseconds was also observed. From Fig. 2 this drop of intensity can be estimated to be about 10% of the initial intensity during the first 6.5 ps. This intensity drop was not accompanied by significant changes in the radial or azimuthal peaks size.

In order to obtain the characteristic timescales, results of our measurements were fitted with exponential functions. For the lower IR laser intensity, the Bragg peak integrated intensity was fitted with one exponential function. For the two higher IR laser intensities, the Bragg peak intensity decay could not be fitted with a single exponential function due to the fast drop during the first picoseconds. Therefore, these data were fitted with the two exponential functions which took into account both short and long characteristic timescales. For the radial and azimuthal peaks sizes (FWHM) fitting was performed by a single exponential function for all IR intensities. The results of the fits are presented in Fig. 2 by red lines and show a good agreement with the experimental data. For all three IR laser intensities the exponential fit of Bragg peak parameters such as intensity and peaks size (FWHM) provided characteristic timescales of about 300–400 ps (see Fig. 2 and Table 1). For two higher IR laser intensities the short timescale on the order of 5 ps was also revealed by the analysis of the Bragg peak intensities.

### Physics of high-power laser interaction with matter

To further analyze the obtained scattering results, we propose the following model of the IR laser-matter interaction (see Fig. 1.). At the first stage the incoming high-power IR laser pulse ionizes the top layer of colloidal particles, creating a confined plasma on the top of the colloidal crystal. The processes of plasma creation and dynamics are modeled taking into account the periodicity of the colloidal crystal sample and the polystyrene properties and will be discussed below in this section.

Polystyrene is a dielectric material and has no free electrons in the ground state. It is also known to be transparent for the incoming IR pulses with 1.55 eV energy at low laser intensities. However, the situation changes dramatically at high IR laser intensities. The tight focusing of a 50 fs IR laser pulse with the energy about millijoules produces an IR laser intensity on the order of hundreds of terawatts per square centimeter. At such high IR laser intensities the so-called field ionisation is important. It causes the plasma formation in the top layer of the colloidal crystal which may be described by the Keldysh theory^42^. An important parameter of the theory is the so-called Keldysh parameter *γ*,

1 $$\gamma =\frac{{\omega }_{IR}\sqrt{2{m}_{e}{E}_{i}}}{E}=\{\begin{array}{c} > 1\to {\rm{m}}{\rm{u}}{\rm{l}}{\rm{t}}{\rm{i}}-{\rm{p}}{\rm{h}}{\rm{o}}{\rm{t}}{\rm{o}}{\rm{n}}{\rm{r}}{\rm{e}}{\rm{g}}{\rm{i}}{\rm{m}}{\rm{e}},\\  < 1\to {\rm{q}}{\rm{u}}{\rm{a}}{\rm{s}}{\rm{i}}-{\rm{s}}{\rm{t}}{\rm{a}}{\rm{t}}{\rm{i}}{\rm{c}}{\rm{r}}{\rm{e}}{\rm{g}}{\rm{i}}{\rm{m}}{\rm{e}},\end{array}$$

where *ω*_IR_ is the frequency of the laser field, *m*_*e*_ is the electron mass, *E*_*i*_ is the zero-field ionisation energy of an atom, and *E* is the electric field generated by the laser. For low fields and high frequencies (*γ* > 1) the multi-photon ionisation is dominant, while for strong fields and low frequencies (*γ* < 1) the tunneling ionisation prevails. Dependence of the Keldysh parameter on the IR laser intensity is shown in the Supplementary Materials Fig. S3. For all three IR laser intensities and low ionisation states of carbon and hydrogen the Keldysh parameter is lower than unity (see Fig. S3), so the quasi-static ionisation regime prevails^43,44^. The high-intensity IR laser field ionizes atoms in the polystyrene colloidal crystal up to H^+^ and C^+^ for the lower IR intensity and up to C^2+^ for the two higher IR laser intensities used in our experiment (see Supplementary Materials). As a result, plasma is created on top of the colloidal crystal during the first femtoseconds of the IR laser pulse propagation (see Fig. 1b.).

Due to the plasma creation, the incoming IR radiation is not penetrating any more into the colloidal crystal, due to the well-known plasma skin effect^45,46^. The depth of the skin layer *l*_*skin*_ depends on the frequency of the IR laser *ω*_IR_ and plasma frequency ${\omega }_{p}=\sqrt{{n}_{e}{e}^{2}/{m}_{e}{\varepsilon }_{0}}$, where *n*_*e*_ is the number density of electrons, *e* is the electric charge, and *ε*_0_ is the permittivity of free space. In our case, assuming single ionisation of each atom, the skin depth ${l}_{skin}=c/\sqrt{({\omega }_{p}^{2}-{\omega }_{IR}^{2})}$ is about 10–20 nm. Free electrons formed in the skin layer by the strong laser-matter ionisation process are further accelerated by the inverse bremsstrahlung^47^ and resonance absorption^48^ mechanisms. As such, high-energetic electrons are propagating inside the first layer of the colloidal particles of the crystal. Accelerated electrons collide inelastically with the atomic ions inside the colloidal particle which causes additional collisional ionisation of the C atoms up to C^4+^ (see Supplementary Materials for further details). Ionisation and IR laser energy absorption processes described above occur within the colloidal crystal which has a periodic structure. As a result, created plasma also has the same periodicity as the colloidal crystal during the first picoseconds after the IR laser pulse interaction with a colloidal crystal.

In order to reveal the physical processes during the first stages of creation and dynamics of the periodic plasma we used PIConGPU software package (see Methods section for details)^49,50^. These simulations were performed in the time interval from 0 to 1 ps for all three IR laser intensities measured in our pump-probe experiment. Two different types of ionisation processes are dominating in the colloidal crystal, namely field ionisation and collisional ionisation, and they were included in the simulations. The ionisation rate of the field ionisation process was calculated according to the Ammosov-Delone-Krainov (ADK) model^51^ and the collisional ionisation was simulated using the Thomas-Fermi ionisation model^52^ (see Supplementary Materials for details).

**Figure 3:**
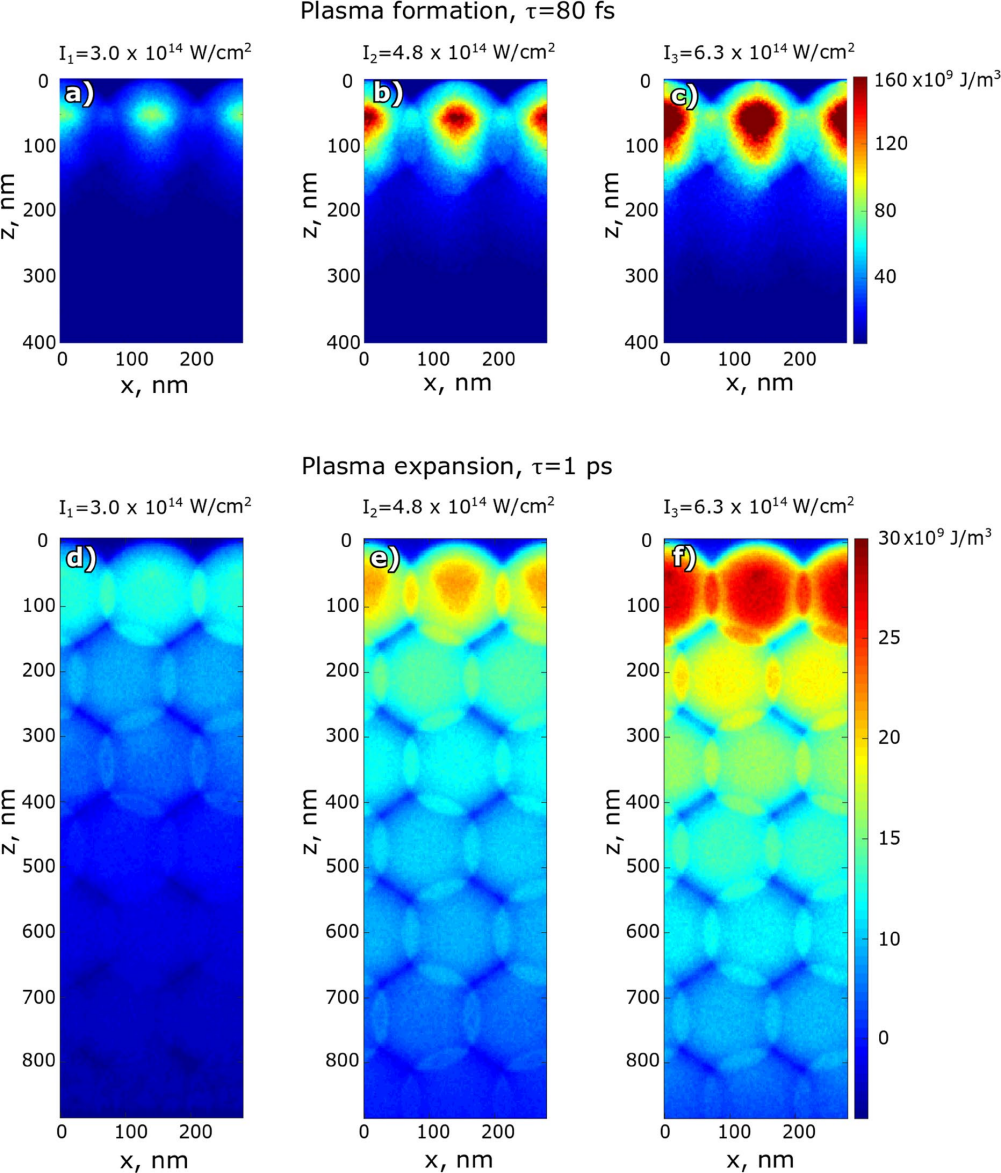
Electron energy density distribution in the colloidal crystal at 80 fs (**a**–**c**) and 1 ps (**d**–**f**) after the start of the IR laser pulse propagation for three different IR laser intensities. The IR laser pulse is coming from the top along the *z*− direction. Here, we show a projection of the electron energy density along the y-direction.

The electron energy density distribution as a function of depth and transverse spatial coordinate at 80 fs and 1 ps after the start of the IR laser pulse propagation are shown in Fig. 3. As one can see from Fig. 3(a–c), at 80 fs only the first layer of the colloidal particles is strongly ionized by the IR laser pulse. As a result, the periodic plasma is formed on top of the colloidal crystal. Our simulations show that the maximum electron energy density was reached in the center of colloidal particles of the first layer 80 fs after the start of the IR laser pulse propagation into the crystal (see Fig. 3(a–c)). The maximum electron energy density is summarized in Table 2 for three measured IR laser intensities. The pressure reaches its maximum in the first layer in the center of each colloidal particle, thus forming a periodic plasma state.

**Table 2:** Results of the plasma and shock wave simulations. Maximum electron energy density was obtained from the PIConGPU simulations. Ablation depth, shock wave parameters, and maximum mass velocity were obtained from the HELIOS simulations.

Intensity, 10^14^ W/cm^2^	3.0	4.8	6.3
Maximum electron energy density at 80 fs (see Fig. 4), 10^9^ J/m^3^	49	98	149
Maximum electron energy density at 1 ps (see Fig. 4), 10^9^ J/m^3^	12	20	25
Ablation depth at 1 ns (see Fig. 5), nm	180	280	450
Shock wave stop times (see Fig. 5), ps	437	756	931
Shock wave depth at the stop time (see Fig. 5), *μ*m	2.36	4.15	5.00
Maximum mass velocity (see Fig. S11), km/s	2.3	2.7	2.8

At 1 ps after the beginning of the interaction of the IR laser with the colloidal crystal sample, accelerated electrons move deep inside the colloidal crystal and collisionally ionize the inner part of the crystal (see Fig. 3(d–f) and Supplementary Materials for further details). The electron energy density at 1 ps has its maximum still in the first layer of the colloidal crystal but its magnitude is much lower than at 80 fs (see Table 2). Even after 1 ps the electron energy density distribution resembles a periodic structure of the colloidal crystal (see Fig. 1c.).

**Figure 4:**
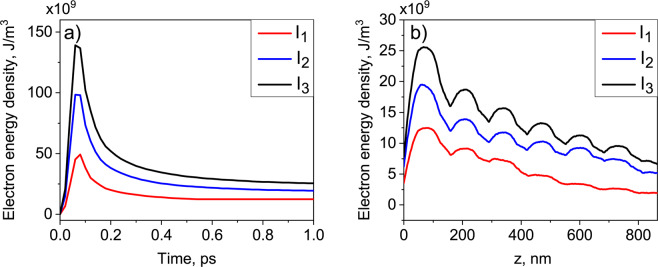
Time (**a**) and depth (**b**) dependencies of the electron energy density for three measured intensities *I*_1_ = 3.0 · 10^14^ W/cm^2^, *I*_2_ = 4.8 · 10^14^ W/cm^2^ and *I*_3_ = 6.3 · 10^14^ W/cm^2^. Time dependencies of the electron energy density are shown at the depth of 75 nm from the top of the sample that corresponds to the center of colloidal particles in the first surface layer. Electron energy density-depth dependence is shown at 1 ps after the start of interaction with the IR laser pulse.

To determine the evolution of the electron energy density distribution, we averaged simulated values over the transverse coordinates. The time dependence of the maximum electron energy density is shown in Fig. 4(a). As shown in this figure, the electron energy density reaches its maximum value at 80 fs for all IR laser intensities values, and after 0.6 ps remains practically constant. The z-dependence at 1 ps is shown in Fig. 4(b). It is clearly seen that the electron energy density is decaying along the z-direction, but periodic modulations due to the colloidal crystal structure are well visible. This periodicity is less prominent for the lower IR laser intensity because of the low ionisation level of the inner part of the colloidal crystal.

### Ablation and shock wave propagation

After the plasma formation, the electron-ion thermalisation occurs and further dynamics in the colloidal crystal is governed by the hydrodynamics. These processes are ablation of the material and shock wave propagation inside the cold material. This last hydrodynamic stage of our model, which was also observed in our experiment, will be discussed in this section.

As it can be seen from Fig. 4(a), the ionisation of the colloidal crystal was practically finished at 1 ps. Around these times the high-pressure dense plasma in the top layers of the colloidal crystal induced ablation and shock wave propagation inside the sample (see Fig. 1d.). As a result, the top layers of the sample were ablated and a strong shock wave compressed the solid and destroyed the periodicity of the inner part of the colloidal crystal. We relate the experimentally observed fast drop of the scattered intensity to the ablation process of the top layers of the colloidal crystal. The ablation of different materials as a result of interaction of high-power IR lasers and matter has been intensively studied, both experimentally and theoretically^53–55^. The shock wave propagation manifests itself as an exponential drop of intensity with the typical time scales on the order of hundreds of picoseconds (see Fig. 2) in the IR pump–X-ray probe diffraction experiment.

**Figure 5:**
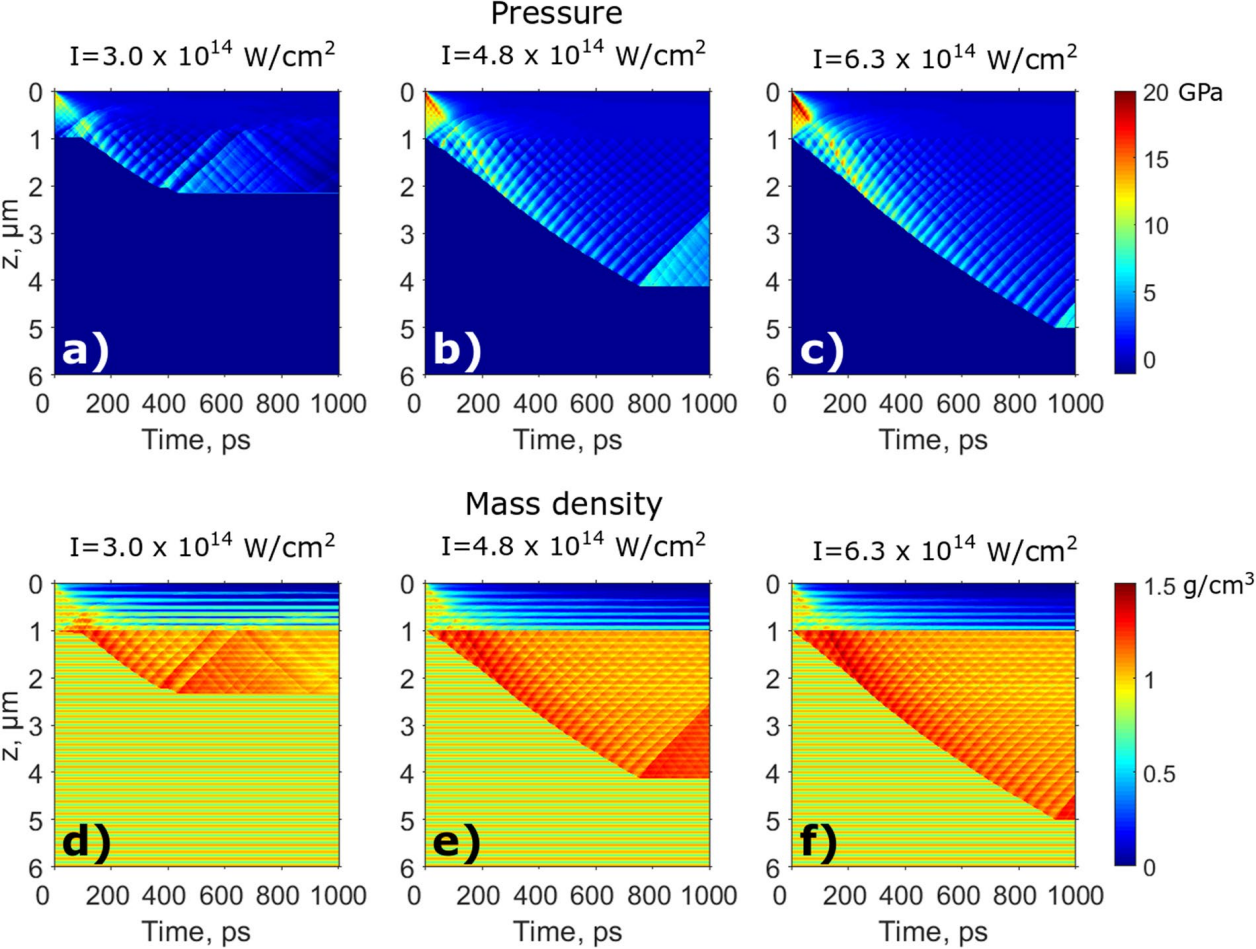
Hydrodynamic simulations of the shock wave propagation. Color plots show simulation results for the pressure (**a**–**c**) and mass density (**d**–**f**) for three different IR laser intensities: (**a**,**d**) *I*_1_ = 3.0 · 10^14^ W/cm^2^, (**b**,**e**) *I*_2_ = 4.8 · 10^14^ W/cm^2^, (**c**,**f**) *I*_3_ = 6.3 · 10^14^ W/cm^2^.

In order to model structural changes in the colloidal crystal, hydrodynamic simulations using the HELIOS software package were performed^56^ (see Methods for simulation details). This software package is widely used to simulate the dynamics of plasma evolution created in high-energy density physics experiments^2,11,34^. The HELIOS code is adapted for simulations in one dimension, therefore the 3D structure of the colloidal crystal was modeled as layers with the periodic variation of a mass density. The simulations were performed using the two-temperature model, which takes into account the fact that the energy of hot electrons is not instantaneously transferred to cold ions, but is governed by the electron-phonon coupling. The pressure and mass density evolution obtained from the hydrodynamic simulations are shown in Fig. 5 (see also Supplementary Materials).

The first process that was determined in the hydrodynamic simulations is the ablation of the material on the top of the colloidal crystal. The ablation threshold of polystyrene for 800 nm laser wavelength with 40 fs pulse duration as reported in^57^ is on the order of 10 mJ/cm^2^. The laser fluences used in our pump-probe experiment were three orders of magnitude higher than the polystyrene ablation threshold (see Table 1). During the first picoseconds the top layers of the colloidal crystal were already damaged by the ablation process, and we observe a steep gradient of the mass density in our hydrodynamic simulations (see Fig. 5(d–f), and Supplementary Materials for details). After the first picoseconds, the ablation process continues and results in a zero mass density on the top of the sample. Due to the ablation process of the top layers of the colloidal crystal we observed a fast initial drop of the scattered intensity in Fig. 2(b,c). The ablation process stops at about 180 nm – 450 nm depth which corresponds to about 1–3 layers (see Table 2).

The next process that occurs is the shock wave propagation inside the periodic colloidal crystal. As one can see from Fig. 5(a–c), the shock pressure is propagating along the z-direction and destroys the periodicity of the significant part of the sample. During the first picoseconds the maximum shock wave pressure is located in the top layer of the colloidal crystal (see Figs. 4, 5(a–c)). The shock wave speed is proportional to the square root of pressure, and it was about 6 km/s on the top of the sample and about 4 km/s on the border of the shock wave front with the cold material. Therefore, at a times of 100 ps and the depths of one micrometer (that corresponds to about 8 layers) the high pressure front reaches the low pressure front (see Fig. 5 and Supplementary Materials). After 100 ps the high pressure front propagates further inside the sample and destroys the sample periodicity. The average shock wave propagation speed obtained from our simulations is on the order of 5 km/s and maximum mass velocity is on the order of 2 km/s which is in a good agreement with previous studies^58^.

While propagating inside the colloidal crystal, the shock wave is losing its speed due to dissipation of energy and consequently the pressure of the shock wave front is decreasing gradually. At the distance where the shock wave pressure is not sufficient to compress the colloidal crystal, the shock wave effectively stops. For three IR laser intensities used in this experiment, the shock wave stopped after approximately 400 ps – 900 ps propagation time at a depth of 2 *μ*m–5 *μ*m and these values are provided in Table 2. The sample periodicity was not further destroyed beyond this point, because the shock wave converts into a sound wave, which does not induce any structural transformation of the colloidal crystal sample^59^.

In order to study the influence of the sample periodicity on the shock wave propagation, we performed hydrodynamic simulations for the non-periodic polystyrene sample. The results were obtained for all three IR laser intensities used in our XFEL experiment and are summarized in Supplementary Materials. From the comparison of these two sets of simulations we can conclude that for the non-periodic sample the shock wave stops earlier and the depth of the shock wave propagation is about 30% smaller than for the periodic one. The ablation depth is also about twice shorter for the non-periodic sample (see Supplementary Materials Figs. S15–S17 and Table SIV). Such difference can be explained by the higher average mass density in the case of the non-periodic sample. A comparison of two simulation sets shows that the pressure modulations observed in Fig. 5(a–c) are caused by the periodic structure of the colloidal crystal sample.

## Discussion

In the present work we studied experimentally and by theoretical modelling the dynamics of the periodic plasma induced by high-power IR laser in the polystyrene colloidal crystals. We performed a pump-probe diffraction experiment at LCLS on a periodic plasma created from a colloidal crystal sample. The periodic structure of the colloidal crystal allowed us to measure Bragg peaks from the sample. We observed a fast decay of the Bragg peak intensity and the growth of the radial and azimuthal peaks width. Such changes of scattering parameters indicate ultrafast dynamics of the colloidal crystal periodic structure. From the analysis of the Bragg peak parameters we obtained 5 ps short and 300 ps long characteristic timescales.

We have proposed a three-stage model of interaction between the high-power IR laser pulse and the periodic colloidal crystal to explain the ultrafast changes in the colloidal sample (see Fig. 1). First, all colloidal particles are in the initial condition, unaffected by the laser pulse. The incoming IR laser pulse generates a plasma in the top layer of the colloidal crystal within the first few femtoseconds (see Fig. 1(a,b)). Due to the plasma skin effect the IR laser pulse is partially reflected (see Fig. 1(b)). This hot confined periodic plasma then expands inside the colloidal crystal up to about 0.6 ps time (see Fig. 1(b)). The second stage is the ablation of a few layers on the top of the colloidal crystal. The top layers of the sample are damaged during the first few picoseconds and are completely destroyed afterwards (see Fig. 1(d)). During the third and last stage the shock wave is formed and propagates inside the colloidal crystal sample. This shock wave destroys the periodicity of the sample by compressing the structure in its deeper parts (see Fig. 1(d)).

The three-stage model of the laser-matter interaction allowed us to attribute the short 5 ps time scale determined in our diffraction pump-probe experiment to the ablation of the material. The long 300 ps time scale is related to the shock wave propagation. Simulations were performed for all three stages of the laser-matter interaction: plasma formation and expansion were simulated with the 3D PIConGPU code, ablation and shock wave propagation were simulated with the HELIOS code. From the results of plasma and hydrodynamic simulations the time dependence of the structural changes in the colloidal crystal was obtained. The results of simulations are in a good agreement with the analysis of the Bragg peaks, extracted from the diffraction patterns measured in our pump-probe experiment (see Fig. 2, Tables 1 and 2)). In the hydrodynamic simulations, the shock wave stops at different depth of the colloidal crystal depending on the incoming IR laser intensity (see Fig. 5 and Table 2). As a result, the amount of colloidal crystal affected by the shock wave is higher for higher IR laser intensity and this is consistent with the experimental data.

Finally, we demonstrated that the shock wave propagation inside the periodic colloidal crystal can be visualized *in situ* with a high temporal resolution by an IR pump – X-ray probe experiment at an XFEL facility. The periodic structure of the colloidal crystal allowed us to reveal the picosecond dynamics of the propagating shock wave by Bragg peak analysis. We obtained short and long characteristic timescales corresponding to the ablation of the material and shock wave propagation, respectively. At the same time our simulations predict much shorter times of evolution of plasma and ablation processes in polystyrene colloidal samples. This is still an open and intriguing question of investigation of the plasma dynamics and ablation process with the sub-picosecond time resolution and will need special attention in future experiments.

By performing IR-pump and X-ray probe experiments on the periodic samples we foresee that formation and development of the periodic plasma may be studied in detail in future. The application of these ideas and methodology based on scattering from the periodic samples may lead towards new ways of investigating of phase transitions in matter under extreme conditions.

## Methods

### Experiment

The pump-probe experiment was performed at the Linac Coherent Light Source (LCLS)^12^ in Stanford, USA at the X-ray Pump Probe (XPP) beamline^39^ (see also for the details of experiment^40^). LCLS was operated in the Self Amplified Spontaneous Emission (SASE) mode. We used LCLS in the monochromatic regime with the photon energy of a single XFEL pulse of 8 keV (1.5498 Å), energy bandwidth Δ*E/E* of 4.4 · 10^−5^, and pulse duration of about 50 fs at a repetition rate of 120 Hz.

The X-ray beam was focused using the Compound Refractive Lenses (CRL) on the sample down to 50 *μ*m Full Width at Half Maximum (FWHM). The experimental setup is shown in Fig. 1 and the detailed description is given in^40^. Series of X-ray diffraction images were recorded using the Cornell-SLAC Pixel Array Detector (CSPAD) megapixel X-ray detector^60^ with a pixel size of 110 × 110 *μ*m^2^ positioned at the distance of 10 m and covering an area approximately 17 × 17 cm^2^. Our experimental arrangement provided a resolution of 0.5 *μ*m^−1^ per pixel in reciprocal space.

The Ti:Sapphire IR laser was used to pump the colloidal crystals. The pump pulses were generated at the wavelength *λ* = 800 nm (1.55 eV) and duration of about 50 fs (FWHM). The IR laser pulses were propagating collinear with the XFEL pulses and were synchronized with the XFEL pulses with less than 0.5 ps jitter. The size of the laser footprint on the sample was 100 *μ*m (FWHM) and, therefore, twice the size of the X-ray beam.

The pump-probe experiment was conducted in the following way. First, for each position of the sample and each time delay 100 diffraction patterns without the IR laser were measured. Next, 100 diffraction patterns with the IR laser and a fixed time delay were measured. The fast CSPAD detector allowed us to determine the diffraction pattern just after the first irradiation by the IR laser pulse. For two lower IR laser intensities five diffraction patterns were measured at a new positions of the sample for each time delay. For higher IR laser intensity, the measurements were repeated nine times for the 6.5 ps time delay and 10 times for the 25.25 ps time delay. Each measurement was performed at a new position of the sample as in previous cases.

### Data analysis

Due to the varying intensity of each incoming X-ray pulse, normalization of the diffraction patterns by the incoming beam intensity was necessary. In order to obtain more careful FWHM characterization of the Bragg peaks, projections on the azimuthal and radial directions were performed. These data were fitted with the one-dimensional Gaussian functions and the integrated intensity as well as broadening in the radial and azimuthal directions were determined.

In order to compare the dynamics of the collected data as a function of the time delay *τ*, the following dimensionless parameters were used:

2 $$\frac{\Delta I(\tau )}{I}=\frac{\langle {I}_{on}(\tau )-\overline{{I}_{off}(\tau })\rangle }{\overline{{I}_{off}(\tau )}},$$

3 $$\frac{\Delta w(\tau )}{w}=\frac{\langle {w}_{on}(\tau )-\overline{{w}_{off}(\tau )}\rangle }{\overline{{w}_{off}(\tau )}}.$$

Subscript letters ‘on’ and ‘off’ define measurements with and without the IR laser, respectively. The ‘off’ pulses were averaged over 100 incoming pulses for each time delay. The brackets $\langle \ldots \rangle$ correspond to averaging of the chosen Bragg peak parameter over the different positions at the sample.

### Plasma simulations

To simulate plasma formation in the colloidal crystal during the first picosecond of the IR laser pulse propagation we used Particle-In-Cell on Graphic Processor Units (PIConGPU) code version 0.4.0-dev developed at Helmholtz-Zentrum Dresden-Rossendorf (HZDR)^49,50^ PIConGPU is a fully-relativistic, open-source Particle-in-Cell (PIC) code running on Graphics Processing Units (GPUs). The PIC algorithm solves the so-called Maxwell-Vlasov equation describing the time evolution of the distribution function of a plasma consisting of charged particles (electrons and ions) with a long-range interaction. The simulated volume of the colloidal crystal was considered according to the colloidal particle size (*d* = 163 nm). The simulation box was considered to be 284 × 163 × 1150 nm^3^ in the *x* × *y* × *z* directions with the 2.2 nm cell size in the *x*− and *z*− directions and 2.5 nm cell size in the *y*− direction (see Fig. S1 in Supplementary Materials). On the top and bottom of the simulation box additional absorbing layers were introduced. In the PIConGPU simulations the IR laser wavelength was set to 800 nm. Simulations were performed with a 4.25 attosecond time increment in order to resolve the plasma frequency oscillations.

### Hydrodymic simulations

Shock wave and ablation simulations were performed using 1D HELIOS code solving one-dimensional Lagrangian hydrodynamics equations. We used the two-temperature model option for the hydrodynamic simulations. The PRism OPACity and Equation Of State code (PROPACEOS) was used as an equation of state for polystyrene. The hydrodynamic simulations were coupled to the plasma PIConGPU simulations in the following way. The 1D projection of the electron energy density profile at 1 ps obtained from the PIConGPU simulations was calculated (see Fig. 4). The electron energy density for the initial condition was converted to the electron temperature according to PROPACEOS equation of state (see Supplementary Materials for details). The electron temperature was extended up to 6 *μ*m according to the room temperature conditions. The ions were assumed to have the room temperature. The calculated electron temperature and one-dimensional projection of the density of the hexagonal-close-packed colloidal crystal structure were used as an initial condition of the hydrodynamic simulation.

Hydrodynamic simulations were performed in the time range from 1 to 1000 ps with 1 ps time increment for the 6 *μ*m thick polystyrene colloidal crystals. The boundaries of the plasma were allowed to expand freely. The quiet start temperature was set to 0.044 eV, which is equal to the polystyrene melting temperature. The time increment in the HELIOS simulations was chosen according to Courant condition, and other criteria which constrain the fractional change of various physical quantities used in the simulation. The simulation results were saved each 1 ps due to a large amount of the output data.

## Data Availability

All relevant data are available from the corresponding author.

## Supplemenatry information

Supplemenatry information.
Supplemenatry information2.
