# Supplementary material for: Femtosecond laser produced periodic plasma in a colloidal crystal probed by XFEL radiation
Source: Sci Rep. 2020 Jul 1;10:10780. doi: 10.1038/s41598-020-67214-z (PMC7329833; doi:10.1038/s41598-020-67214-z)
Supplement: Supplementary file 2 — Supplemenatry information2. [file 41598_2020_67214_MOESM2_ESM.pdf]

Femtosecond laser produced periodic plasma in a colloidal crystal probed by XFEL radiation. Supplementary Materials.

Nastasia Mukharamova,[1] Sergey Lazarev,[1,2] Janne-Mieke Meijer,[3,*] Oleg Yu. Gorobtsov,[1,†] Andrej Singer,[4,†] Matthieu Chollet,[5] Michael Bussmann,[6,7] Dmitry Dzhigaev,[1,‡] Yiping Feng,[5] Marco Garten,[6,8] Axel Huebl,[6,8,§] Thomas Kluge,[6] Ruslan P. Kurta,[1,¶] Vladimir Lipp,[9] Robin Santra,[9,10] Marcin Sikorski,[5,¶] Sanghoon Song,[5] Garth Williams,[5,**] Diling Zhu,[5] Beata Ziaja-Motyka,[9,11] Thomas Cowan,[6] Andrei V. Petukhov,[3,12] and Ivan A. Vartanyants[1,13,††]

[1]*Deutsches Elektronen-Synchrotron DESY, Notkestraße 85, D-22607 Hamburg, Germany*

[2]*National Research Tomsk Polytechnic University (TPU), pr. Lenina 30, 634050 Tomsk, Russia*

[3]*Debye Institute for Nanomaterials Science, University of Utrecht, Padualaan 8, 3508 TB Utrecht, The Netherlands*

[4]*University of California, 9500 Gilman Dr., La Jolla, San Diego, CA 92093, USA*

[5]*SLAC National Accelerator Laboratory, 2575 Sand Hill Rd, Menlo Park, CA 94025, USA*

[6]*Institute of Radiation Physics, Helmholtz Zentrum Dresden-Rossendorf, 01328 Dresden, Germany*

[7]*Center for Advanced Systems Understanding (CASUS), Görlitz, Germany*

[8]*Technische Universität Dresden, 01069 Dresden, Germany*

[9]*Center for Free-Electron Laser Science, DESY, D-22607 Hamburg, Germany*

[10]*Department of Physics, Universität Hamburg, 20355 Hamburg, Germany*

[11]*Institute of Nuclear Physics, PAS, Radzikowskiego 152, 31-342 Krakow, Poland*

[12]*Laboratory of Physical Chemistry,*

Department of Chemical Engineering and Chemistry,

Eindhoven University of Technology P.O. Box 513, 5600 MB Eindhoven, Netherlands

[13]*National Research Nuclear University MEPhI (Moscow Engineering Physics Institute),*

Kashirskoe shosse 31, 115409 Moscow, Russia

[*] Present address: Universiteit van Amsterdam, Science Park 904, 1090 GL Amsterdam, The Netherlands

[†] Present address: Cornell University, Ithaca, NY 14850, USA

[‡] Present address: Division of Synchrotron Radiation Research, Department of Physics, Lund University, S-22100 Lund, Sweden

[§] Present address: Lawrence Berkeley National Laboratory, 1 Cyclotron Rd, Berkeley, CA 94720, USA

[¶] Present address: European XFEL, Holzkoppel 4, D-22869 Schenefeld, Germany

[**] Present address: NSLS-II, Brookhaven National Laboratory, Upton, NY 11973-5000, USA

[††] Corresponding author email: ivan.vartaniants@desy.de

I. INFRARED LASER CALIBRATION

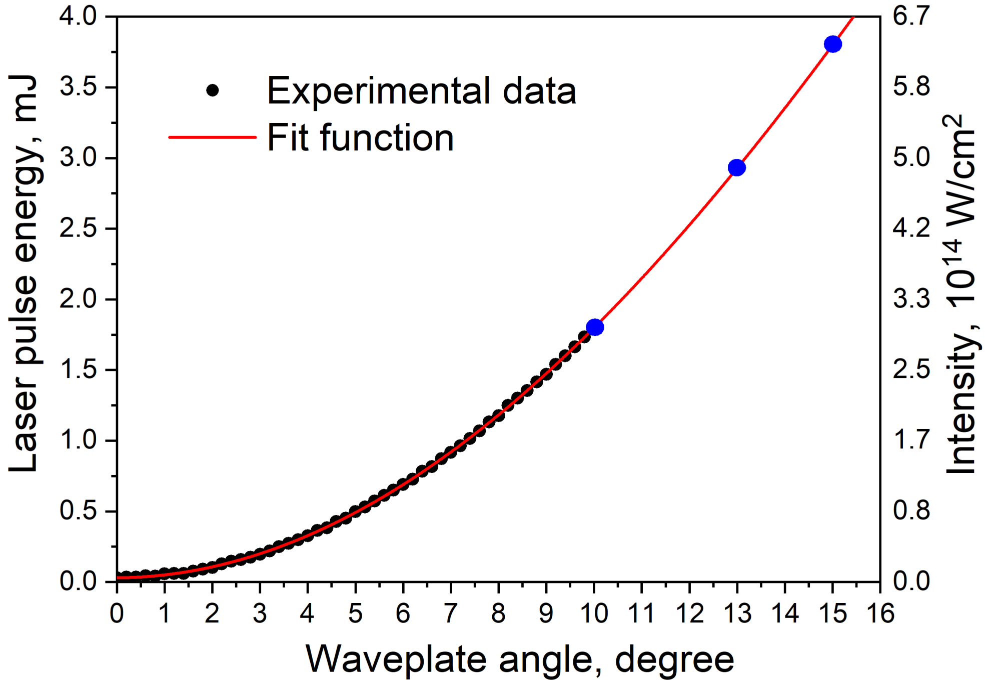

FIG. S1. The infrared (IR) laser energy calibration curve. The measured data is shown by the black dots and the sine fit is shown by the solid red line. The IR laser energy used in the experiments for three samples is marked with blue circles.

In our pump-probe experiment the Ti:sapphire IR laser was used to pump the colloidal crystal film. The IR laser energy was controlled by the rotation of the optical axis of a waveplate, and was calibrated by power sensor at the position of the sample. The calibration curve showing the dependence of the laser pulse energy from the waveplate angle is presented in Fig. S1. The corresponding IR laser intensity is shown on the right vertical axis. The IR laser intensity was calculated from the IR laser energy assuming Gaussian shape of the pulse with 50 fs FWHM in the temporal domain and 100 μm FWHM in the spatial domain. Zero degrees of waveplate angle corresponds to the minimum and 15 degrees correspond to the maximum calibrated energy and intensity of the IR laser. The calibration curve was fitted with a sine function shown by the solid red line in Fig. S1. The three IR laser intensities ($I_1 = 3.0 \cdot 10^{14}$ W/cm^2, $I_2 = 4.8 \cdot 10^{14}$ W/cm^2 and $I_3 = 6.3 \cdot 10^{14}$ W/cm^2) used in the current

experiment are marked by the blue circles. At energies lower than 1 mJ, no ultrafast melting was observed and different dynamics of the colloidal crystal was investigated in a separate work [1].

II. PLASMA FORMATION SIMULATIONS

A. Simulation cell

To simulate plasma formation in the colloidal crystal during the first 1 ps of the IR laser pulse propagation we used PIConGPU code version 0.4.0–dev developed at Helmholtz-Zentrum Dresden-Rossendorf [2, 3]. In the PIConGPU simulation a rectangular shape of the simulation box is considered. The simulated volume of the colloidal crystal was chosen according to the colloidal particle size (d= 163 nm). The simulation box is shown in Fig. S2 and was $284 \times 163 \times 1280$ nm^3 in $x \times y \times z$ directions ($d \times d\sqrt{3}$ in $x \times y$ direction). The top view on the simulation box is shown in Fig. S2(a) and two layers of the hexagonal-close-packed colloidal crystal are shown by red and blue color. From Fig. S2(a) it is clear that such a simulation box is periodic in x and y direction. Therefore, such a size of the simulation box was chosen to apply periodic boundary conditions on the sides (x=0 nm, x = 284 nm and y=0 nm, y=163 nm planes) of the simulation box. On the top and bottom of the simulation box additional absorbing layers were introduced. To optimize the simulation process the simulation box consisted of $128 \times 64 \times 512$ cells in $x \times y \times z$ directions. From that condition, the size of one cell was chosen to be 2.2 nm in x and 2.5 nm in y and z directions. On the top of the simulation box 128 cells or 320 nm were not filled with any colloidal particles. This empty space was introduced in the simulation box to initialize the incoming IR laser.

In order to satisfy Courant Friedrichs Lewy condition [4] the simulations were performed with 4.25 as time increment. Such a time increment allows to resolve the plasma frequency oscillations in the $2.2 \times 2.5 \times 2.5\times$ nm cell size in $x \times y \times z$ directions. The output of the simulation was saved each 5 fs during the first 100 fs and each 20 fs up to 1 ps due to a huge amount of the output data. We used a standard Yee solver scheme [5] and the HDF5 openPMD output [6] implemented in the PIConGPU code.

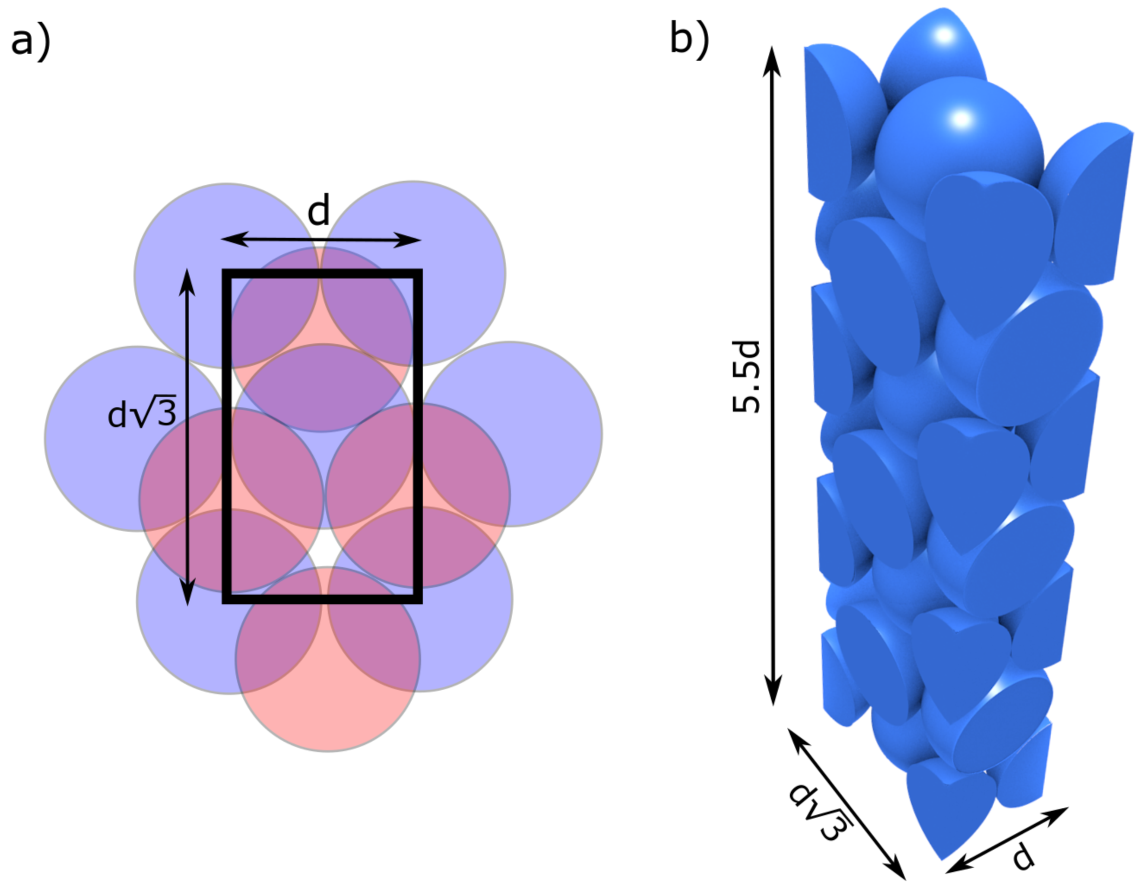

FIG. S2. Simulation cell used in PIConGPU simulations. a) The top view on the simulation box. Different layers of the hexagonal-close-packed colloidal particles are shown in blue and red color. b) 3D view on the simulation box.

B. ADK ionisation of the colloidal crystal

Field ionisation process can be described according to Keldysh theory [7]. For low fields and high frequencies the Keldysh parameter $\gamma > 1$ and multi-photon ionisation happens while for strong fields and low frequencies $\gamma < 1$ and tunneling ionisation prevails. Dependence of the Keldysh parameter on the IR laser intensity is shown in Fig. S3(a). It is clearly seen that for three IR laser intensities used in our experiment Keldysh parameter is lower than one so quasi-static ionisation regime occurred as is also shown in Refs. [8, 9].

Depending on the electric field E generated by the laser in the quasi-static regime, ionisation can be described as tunneling or above-barrier ionisation (ABI). If laser field energy is higher than the threshold $E_{ABI} = E_i/4Z$, where $(Z-1)$ is ion charge, the ionisation is above

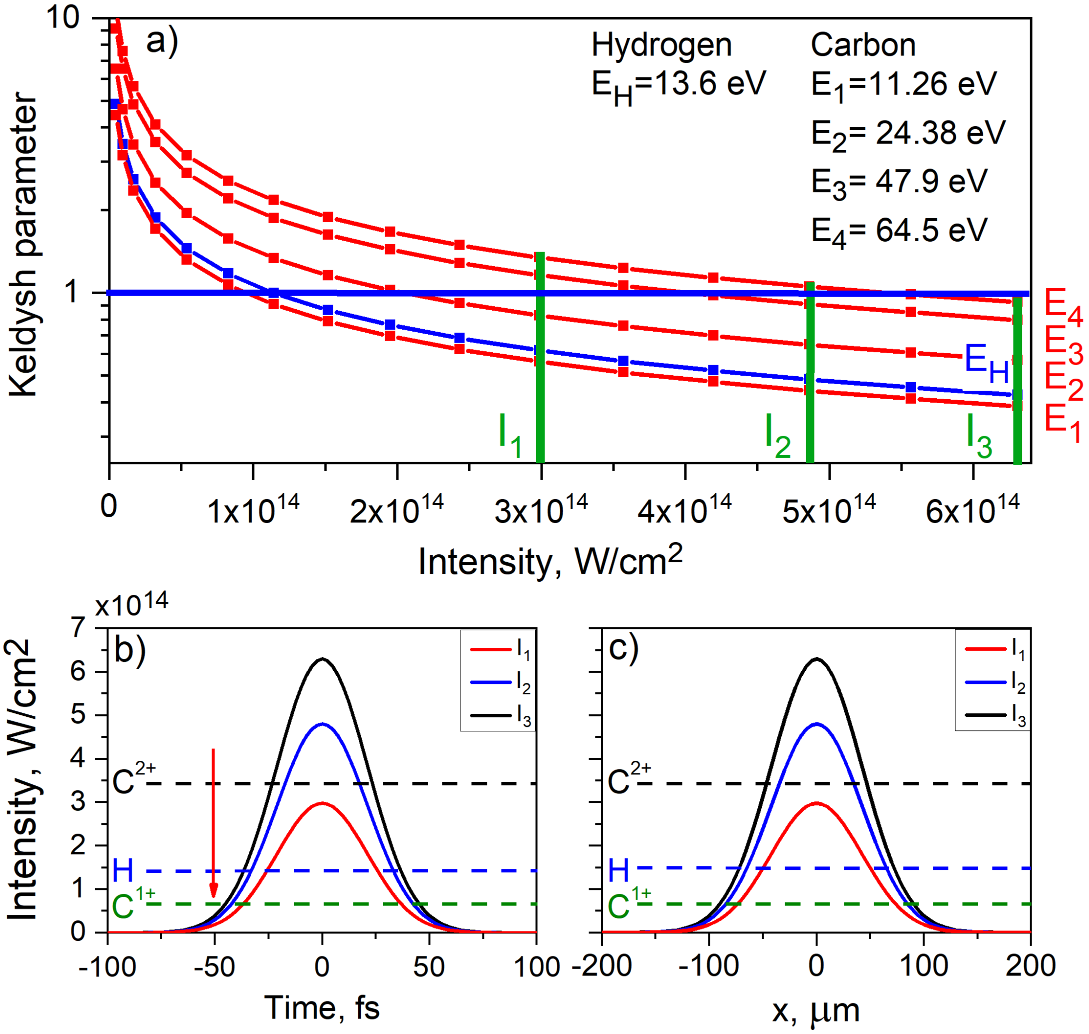

FIG. S3. a) Keldysh parameter for 4 carbon ionisation energies ($E_1 - E_4$) and hydrogen ionisation energy E_H. Measured intensities $I_1 = 3.0 \cdot 10^{14}$ W/cm^2, $I_2 = 4.8 \cdot 10^{14}$ W/cm^2 and $I_3 = 6.3 \cdot 10^{14}$ W/cm^2 are shown by green vertical lines. For C^1+, C^2+ and H^+ Keldysh parameter is lower than one for all three intensities. Temporal b) and spatial c) Gaussian profile of the IR laser. ionisation thresholds for C and H are indicated by horizontal dashed lines. Beginning of the simulation is indicated as vertical red arrow.

barrier and if it is lower the tunneling ionisation is prevailing [10]. ionisation thresholds for ABI are shown at the temporal and spatial profiles of the IR laser intensity in Fig. S3(b,c) by horizontal dashed lines. Beginning of the simulation time is indicated in Fig. S3(b) by the vertical red arrow 50 fs before the IR laser pulse reaches it's maximum. It is well seen that at the beginning of the simulation the IR laser intensity is below ionisation thresholds

for C and H.

From Fig. S3(b,c) it is well seen that for lower laser intensity of $3 \cdot 10^{14}$ W/cm^2 only C^{1+} and H^{1+} are expected to be fully ionized. For two higher laser intensities we also have ionisation of C^{2+}. It is interesting to look also at the spatial distribution of the IR laser pulse (see Fig. S3(c)). In the area of about 150 μm the sample is ionized to C^{1+} and H^{1+} for lower laser intensity, and to C^{2+} for two higher IR laser intensities. Therefore in the 50 μm focused x-ray beam the plasma was considered to be ionized.

The ionisation rate Γ implemented in PIConGPU code was calculated according to Ammosov-Delone-Krainov (ADK) model [11] in the case of a linearly polarized field (an s-state is taken for simplicity) as

$$\Gamma_{ADK} = \sqrt{\frac{3n^{*3}E}{\pi Z^3}} \frac{E}{8\pi Z} \left(\frac{4eZ^3}{En^{*4}}\right)^{2n*} \exp\left(-\frac{2Z^3}{3n^{*3}E}\right) , \qquad (1)$$

where $n^* = Z/\sqrt{2E_i}$ is the effective principal quantum number. The ionisation equations are only in this form if you use the atomic unit system. The ionisation probability is calculated from the ionisation rate as $P = 1 - e^{-\Gamma_{ADK}\Delta t}$.

The version of the ADK model that was used in the PIConGPU code is simplified due to the fact that our particles are carbon and hydrogen and do not have much inner structure. This model was applied for both the tunneling regime $E < E_{ABI}$ and above-barrier regime $E \approx E_{ABI}$. For strong fields $E > E_{ABI}$ where the potential barrier binding an electron is completely suppressed the so-called barrier-suppression ionisation (BSI) regime is reached. Therefore, the ADK model was combined with a check for the BSI threshold. Also the ADK model used in this work is based on the assumption that the material investigated consists of independent atoms and it did not consider the molecular-orbital structure of covalently bonded materials.

In Fig. S4 ionisation rates calculated according to ADK ionisation model for H, C^{1+} and C^{2+} are shown. The intensities of the IR laser used in our experiment are marked by the vertical dashed lines. As can be seen from Fig. S4 the ionisation rate is growing with the field strength up to 10^{17} s^{-1} for the C^{1+}. From this figure one can conclude that each carbon and hydrogen atoms are ionized up to C^{1+} and H^{1+} for all three IR laser intensities and for two higher IR laser intensities significant amount of C^{2+} is created.

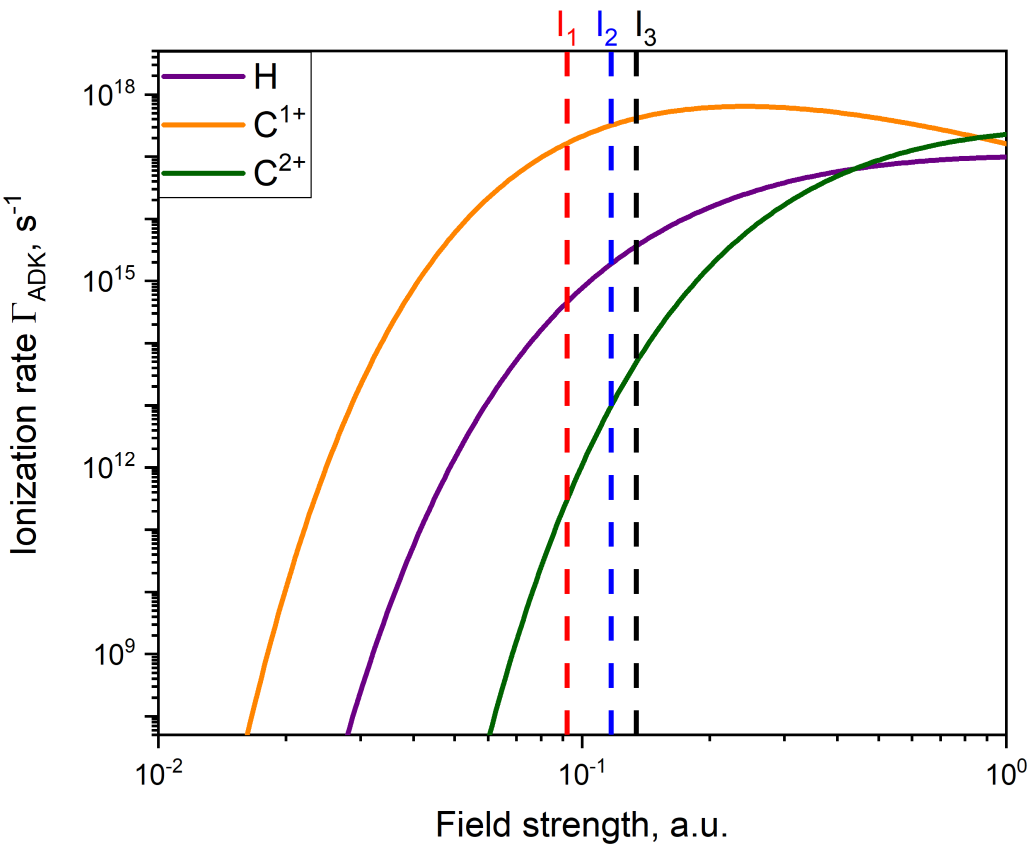

FIG. S4. ADK ionisation rates for three different IR laser intensities. The ionisation rates are shown for H, C^{1+} and C^{2+} by solid lines. IR laser intensities $I_1 = 3.0 \cdot 10^{14}$ W/cm^2, $I_2 = 4.8 \cdot 10^{14}$ W/cm^2, $I_3 = 6.3 \cdot 10^{14}$ W/cm^2 are marked by the vertical dashed lines.

C. Thomas-Fermi ionisation of the colloidal crystal

The second ionisation mechanism is collisional ionisation. Accelerated electrons collide inelastically with the atomic ions inside the colloidal particle which causes ionisation of the atom. Due to the collisional ionisation mechanism, the atoms in the inner part of the colloidal particle are ionized to C^{4+} for the highest IR laser intensity. The ionisation rate of this process was calculated according to the Thomas-Fermi ionisation model [12]. Thomas-Fermi ionisation model uses the self-consistent method, where atom is represented as a point nucleus embedded in a spherical cavity in a continuous background positive charge. The cavity radius R_0, is determined by the plasma density ($\rho = 3M_p/4\pi R_0^2$, where M_p is the

atomic mass). The ionisation state was calculated using an approximate fit to the definition of the ionisation state

$$Z^*(\rho, T) = 4/3\pi R_0^3 n(R_0) \ , \tag{2}$$

where the ionisation state $Z^*(\rho, T)$ is defined from the boundary density $n(R_0)$. The parameters of the fit which were used in PIConGPU can be found in Table 4 in Ref. [12]. The ion proton number, ion species mass density, and electron temperature are used as an input for the Thomas-Fermi ionisation model in the PIConGPU simulation.

The charge state estimates for carbon and hydrogen obtained from this model are shown in Fig. S5. As can be seen from this figure the Thomas-Fermi model displays unphysical behavior in several cases, thus the cutoff values were introduced, to exclude some particles from the calculation. For carbon and hydrogen it predicts non-zero charge states at zero temperature, therefore the lower electron-temperature cutoff value should be defined, and in our model it was 1 eV. For low ion densities Thomas-Fermi model predicts an increasing charge state for decreasing ion densities (see Fig. S5). This occurs already for electron temperatures of 10 eV and the effect increases as the temperature increases. Low ion-density cutoff value was $1.74 \cdot 10^{21}$ ions/cm^3 in our case. Also, super-thermal electron cutoff value was introduced to exclude electrons with kinetic energy above 50 keV. That is motivated by a lower interaction cross-section of particles with high relative velocities.

D. Ionisation simulations

PIConGPU simulations were performed for all three IR laser intensities measured in our pump-probe experiment. PIConGPU simulations intrinsically included field and collisional ionisation discussed in the previous section and thus the averaged charge state of the colloidal crystal was obtained. The average charge state projections along the y-axis after 80 fs and after 1 ps of the IR laser pulse propagation are shown in Fig. S6. The IR laser pulse is coming from the top along the z-direction, and it starts the ionisation of the colloidal crystal sample.

As one can see from Fig. S6(a-c), after 80 fs only the first layer of the colloidal particles is ionized by the IR laser pulse. The highly ionized skin layer of the thickness about 10 nm on top of the first layer of colloidal crystals is also well visible. The average charge state in the skin layer can reach up to 2.9 and is summarized in Table S1. Also, high charge state is reached in the center of the colloidal particles for all three IR laser intensities, and their

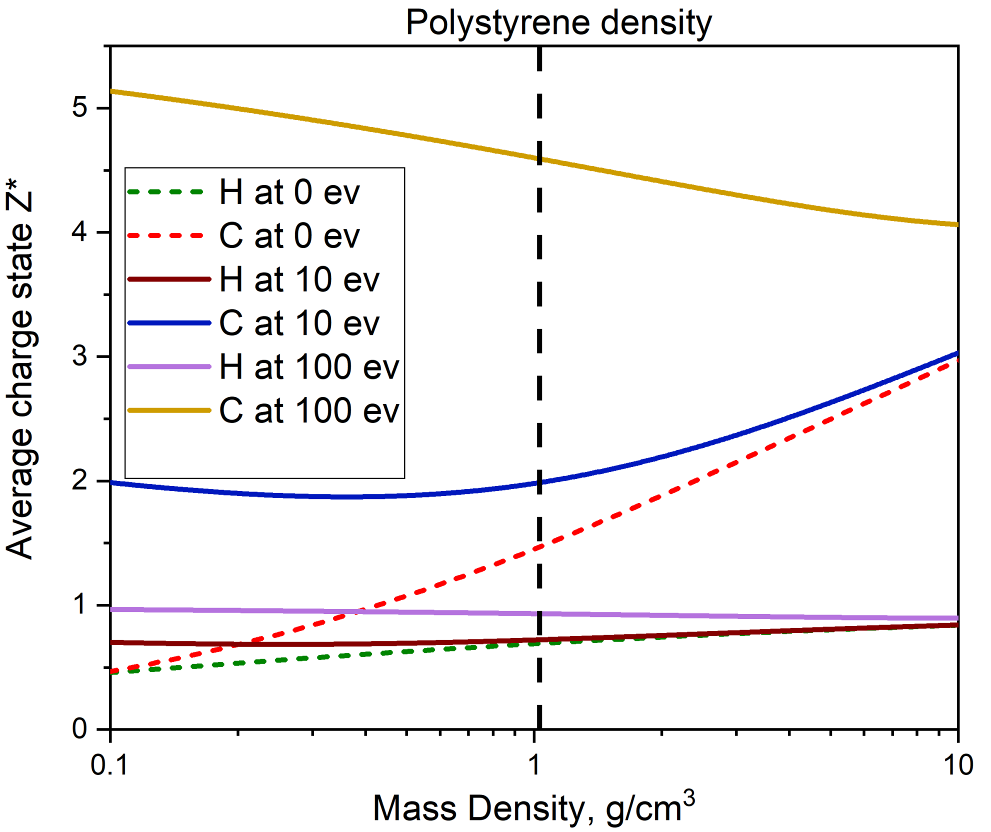

FIG. S5. Thomas-Fermi ionisation for H and C for 0 eV, 10 eV and 100 eV electron temperatures. For 10 eV and 100 eV charge state predictions are marked by solid lines and for 0 eV the charge state predictions are marked by dashed lines because they show unphysical behavior and were excluded from the simulations.

values are summarized in Table S1.

At 600 fs after the beginning of the simulation accelerated electrons collisionally ionized the inner part of the colloidal crystal and until 1 ps the ionisation state of the colloidal crystal remained practically constant (see Fig. S6(d-f) and Supplementary movie). From Fig. S6(d-f) it can be observed that the ionisation depth varies a lot with the IR laser intensity. The ionisation depth is different for three IR laser intensities and is summarized in Table S1. Even at 1 ps after the start of the PIConGPU simulation the highest ionisation state remains at the center of the top layer of the colloidal particles.

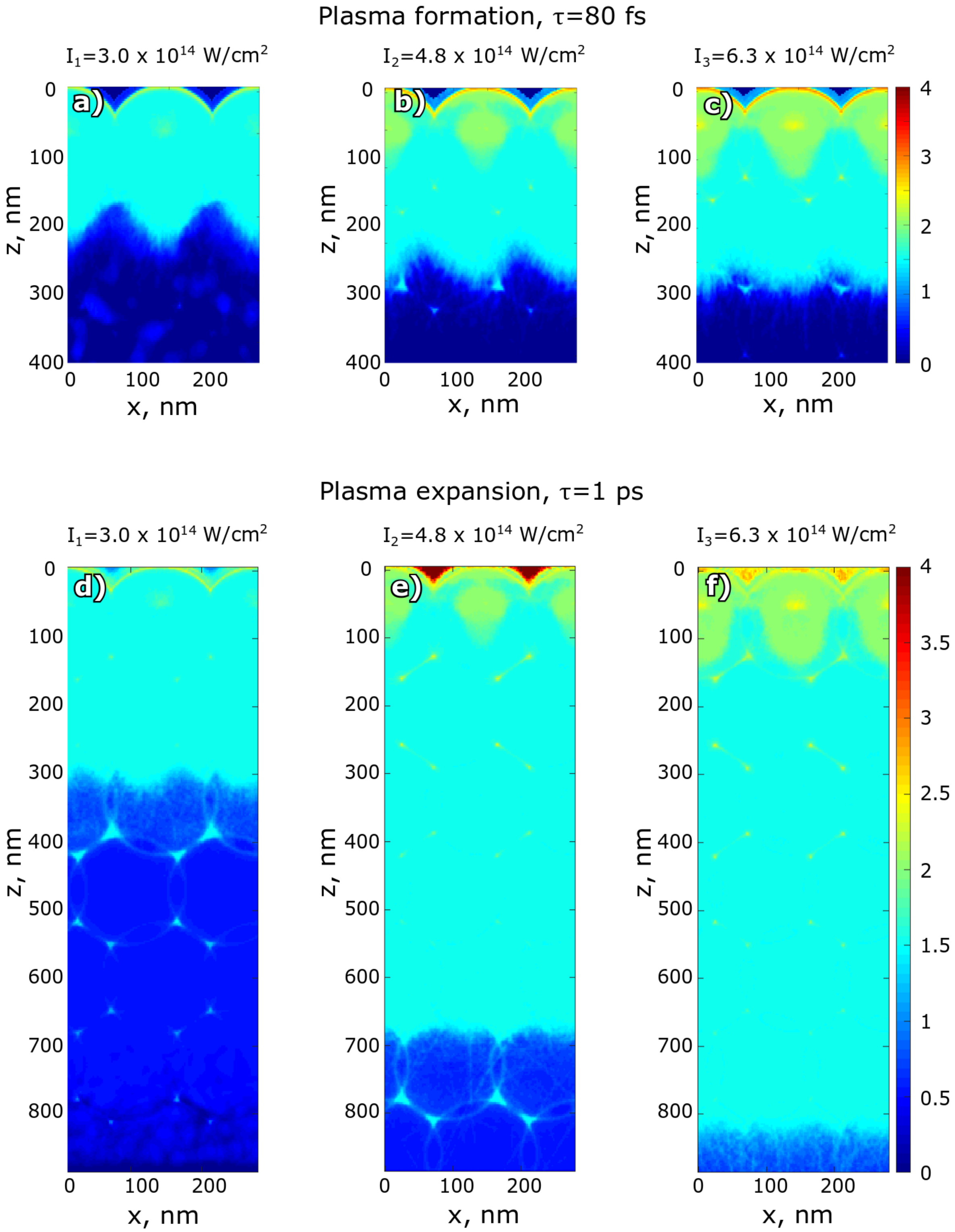

FIG. S6. The average charge state distribution in the colloidal crystal at 80 fs (a-c) and at 1 ps (d-f) after the beginning of the IR laser pulse for three different IR laser intensities. Here we show the projection of the average charge state along the y-direction.

III. HYDRODYNAMIC SIMULATIONS

A. PIConGPU simulations coupled to HELIOS simulations

The HELIOS hydrodynamic simulations were performed to model structural changes in the colloidal crystal due to the shock wave propagation. The hydrodynamic simulations were coupled to the plasma PIConGPU simulations in the following way. The 1D projection of the electron energy density profile at 1 ps was obtained from the PIConGPU simulations. The electron energy density distribution was further converted to the electron temperature using PROPACEOS (PRism OPACity and Equation Of State code) data tables. The horizontal axis is the electron internal energy and the vertical axis is the electron temperature. The PROPACEOS data for polystyrene with typical polystyrene density of 1.05 g/cm^3 is shown by the black dots. This data was fitted with 7th order polynomial function and the polynomial fit is shown by the red line in Fig. S7. Further this polynomial function was used to convert the electron energy density to electron temperature.

The temperature distribution used as an input for hydrodynamic simulations is shown in Fig. S8 for three IR laser intensities. The oscillations of the electron temperature due to the periodic colloidal crystal structure are clearly visible. The PIConGPU simulations were performed for the first 882.5 nm of the colloidal crystal, below this depth up to 6 μm the temperature distribution was set to a room temperature value of 0.025 eV (see Fig. S8). By that we got a drop of the temperature distribution at 882.5 nm depth, which was not smoothed for deeper parts of the colloidal crystal due to the following reasons. The ionisation

TABLE S1. IR laser parameters and results of plasma simulations used in our experiment. The corresponding average ionisation state and ionisation depth were calculated from the PIConGPU simulations.

Intensity, 10^{14} W/cm^2	3.0	4.8	6.3
Average charge state in the skin at 80 fs	2.1	2.7	2.9
Average charge state in the center of the first colloidal particle at 80 fs	1.8	2.1	2.4
Average charge state in the skin at 1 ps	2.1	2.7	2.7
Average charge state in the center of the first colloidal particle at 1 ps	1.7	2.1	2.4
Ionisation depth, nm	300	700	820

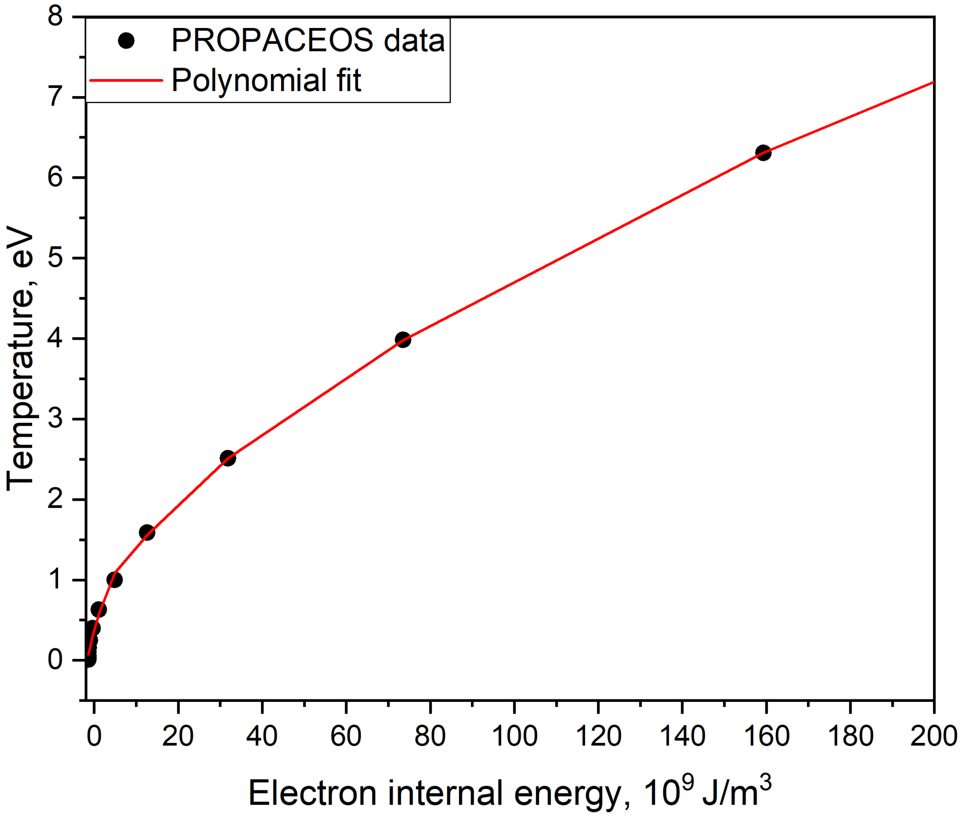

FIG. S7. Polystyrene equation of state obtained from PROPACEOS data tables (black dots) and polynomial fit (solid red line).

rate in the PIConGPU code does not take into account the recombination process, due to that the temperature distribution on the top of the sample should be lower in reality. Our approach was later confirmed by the hydrodynamic simulations performed only by HELIOS code (see section IIIc in Supplementary Materials).

These PIConGPU and HELIOS combined set of simulations is further referred to as a simulation Set 1.

B. Results of the simulation Set 1

The 1D pressure and mass density distribution obtained from the hydrodynamic simulations are shown in Fig. S9, S10, respectively, for all three IR laser intensities. The

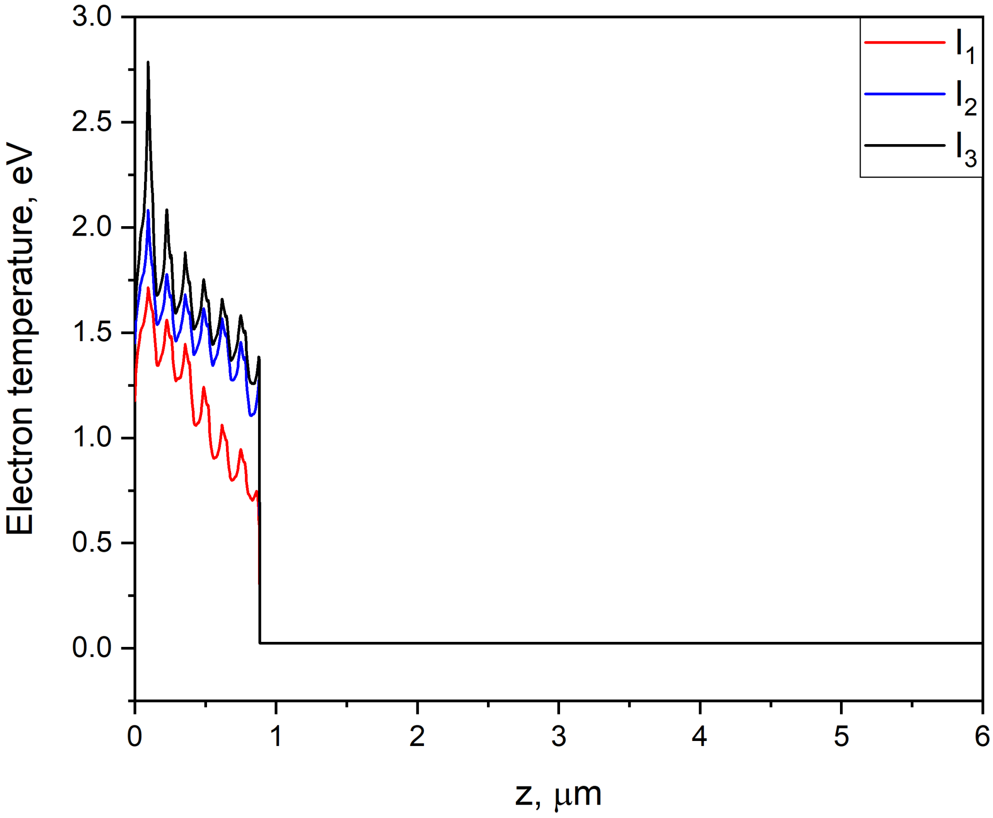

FIG. S8. Electron temperature distribution in the colloidal crystal for three measured intensities $I_1 = 3.0 \cdot 10^{14}$ W/cm^2, $I_2 = 4.8 \cdot 10^{14}$ W/cm^2 and $I_3 = 6.3 \cdot 10^{14}$ W/cm^2 at 1 ps after the IR laser pulse.

initial density distribution used as an input for the hydrodynamic simulations is shown in Fig. S10(a-c). These density profiles were obtained as a projection on the hexagonal-close-packed colloidal crystal along x- and y-direction. The periodicity of the mass density due to colloidal crystal structure is well resolved with the chosen step size of 2.5 nm. The initial pressure distributions are shown in Fig. S9(a-c) as snapshots at 1 ps after the beginning of the IR laser pulse. As one can see at 1 ps the pressure is decaying along the z-direction, and the maximum pressure is in the center of the top layer of the colloidal particles. In the deeper part of the sample the pressure distribution is periodic due to the periodicity of the colloidal crystal. The negative pressures in the simulation are zeroth-order approximation to material strength used in the PROPACEOS and other, for example SESAME EOS tables.

During the first picoseconds of the shock wave propagation the mass density of the approximately 1 μm region on the top of the sample is affected. The snapshot of the mass density at 20 ps is shown in Fig. S10(d-f) where the ablation of the material is clearly visible. The modulations of the mass density become less pronounced and the steep gradient of the mass density going down to zero value is well visible. That is a clear sign of the ablation of the top layer of the colloidal crystal. At 20 ps after the beginning of the HELIOS simulation the high pressure from the top of the colloidal crystal is propagating inside the sample (see Fig. S9(d-f)). At the top 100 nm of the sample pressure is equal to zero due to ablation of the first layer of the colloidal crystal. At about 100 ps the highest pressure is reaching the shock wavefront and the shock wave propagates inside the sample with increased speed.

The shock wave propagating inside the colloidal crystal compresses the surrounding material and from Fig. S10 it is clearly visible that density at the shock wavefront is higher than density after the shock wavefront. When the energy of the shock wave is not sufficient to compress the sample the shock wave stops. The shock wave stops at different times for three IR laser intensities. The pressure distribution at the moment when the shock wave stops is shown in Fig. S9(j-l). The shock wave depth is marked by the blue dashed line. The shock wave stops at different time and depth for all three IR laser intensities (see Table 1 in the main text). The mass density deeper the shock wavefront remains unperturbed while before the shock wavefront is undergoing some small changes even after the shock wave stops (see Fig. S10(j-o)).

The last time point of the hydrodynamic simulation was at 1000 ps and the mass density and pressure snapshots are shown in Fig. S10(m-o) and Fig. S9(m-o). The shock wave did not propagate any deeper inside the colloidal crystal but the mass density on the shock wavefront is smaller than in Fig. S10(j-l) and the pressure front changed its shape significantly. The ablation of the material has also practically finished at that time. From Fig. S10(m-o) it is well seen that the amount of ablated material is higher for higher IR laser intensity, and the values are summarized in the Table 1 in the main text. The ablation threshold is marked by the blue dashed line in Fig. S10(d-f).

The fluid velocity evolution is shown in Fig. S11 for all three IR laser intensities. The fluid velocity of the unperturbed material is equal to zero and the velocity of perturbed material can be positive (moving down along the z-direction) or negative (moving in the opposite direction). On the top of the colloidal crystal the fluid velocity is negative which is a sign

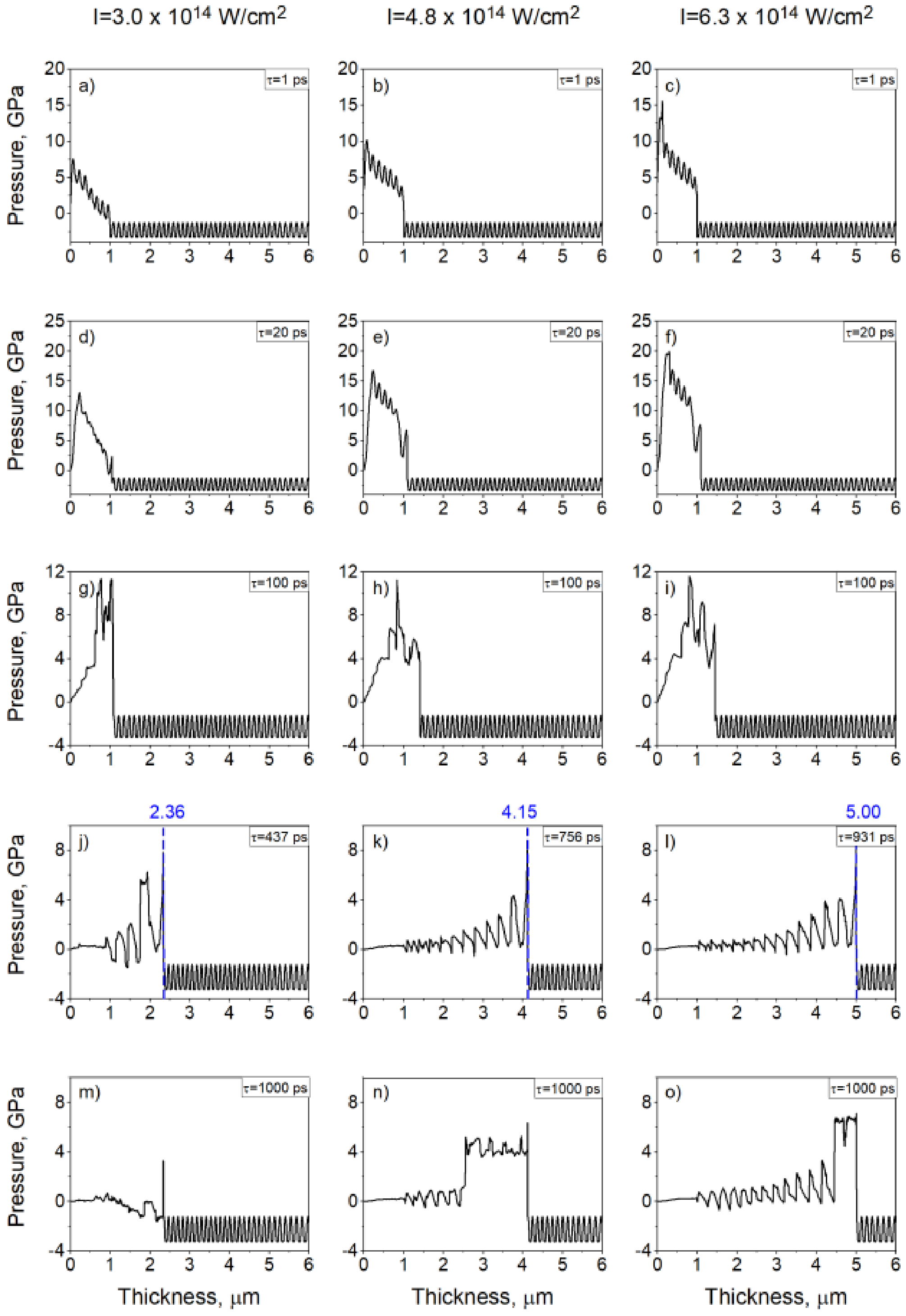

FIG. S9. Pressure in the colloidal crystal from the hydrodynamic simulation. In this case the combination of PIConGPU and HELIOS code was used. Results are shown at 1 ps (a-c), 20 ps (d-f), 100 ps (g-i) after the stop of the shock wave (j-l) and at 1000 ps, the end of the simulation (m-o). The blue dashed line shows the shock wave stop depth (j-l).

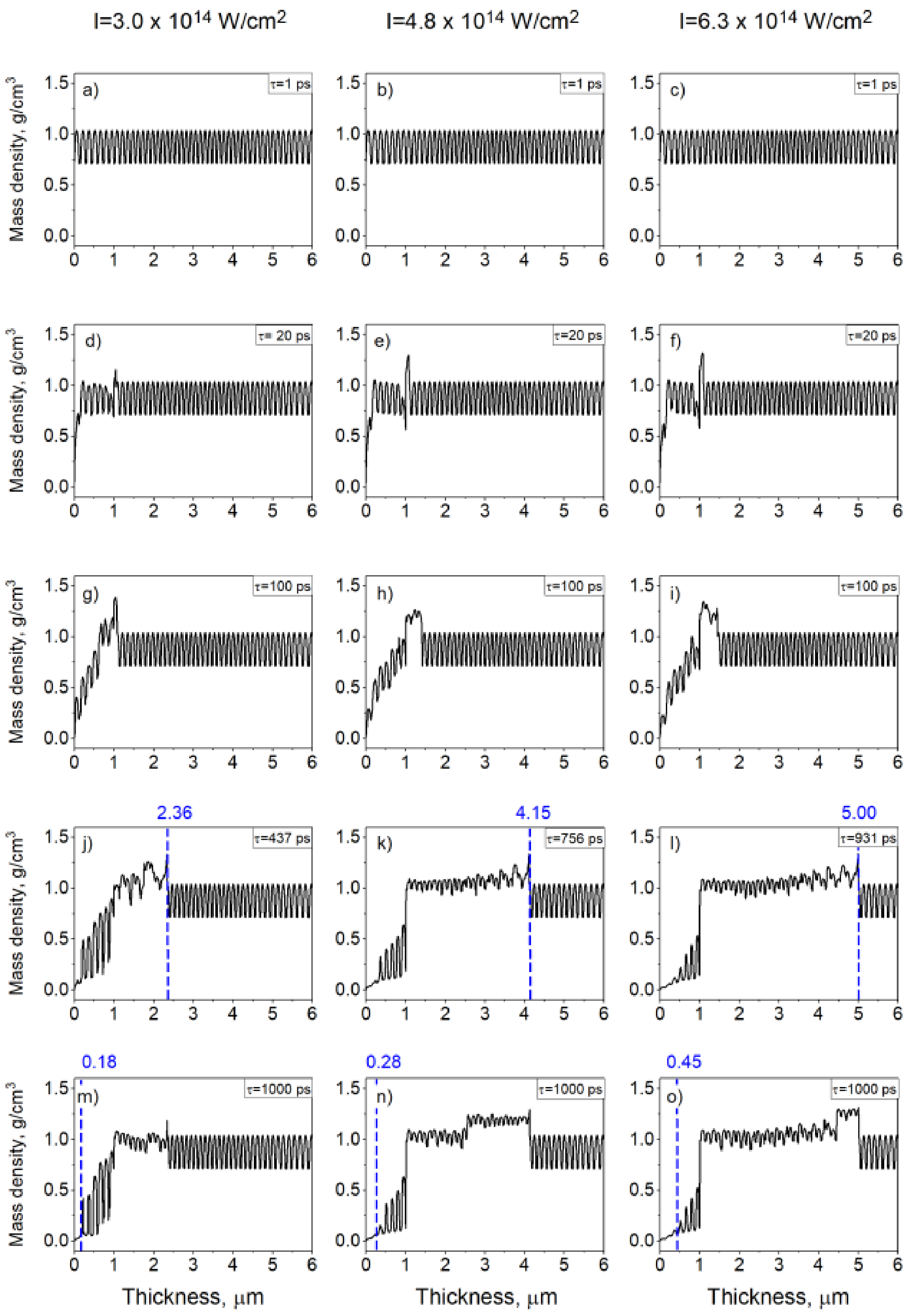

FIG. S10. Mass density of the periodic colloidal crystal from the hydrodynamic simulation. In this case the combination of PIConGPU and HELIOS code was used. Results are shown at 1 ps (a-c), 20 ps (d-f), 100 ps (g-i) after the stop of the shock wave (j-l) and at 1000 ps, the end of the simulation (m-o). The blue dashed line shows the shock wave stop depth (j-l) and the ablation threshold (d-f).

of ablation. On the shock wavefront the velocity is positive, and the liquid polystyrene is moving inside the colloidal crystal. When the shock wave stops the fluid velocity becomes negative due to the reflection of the shock wave. The maximum fluid velocity is observed during the first 100 ps of the shock wave propagation (see Fig. S11). The maximum fluid velocity is on the order of 2 km/s and is summarized in Table 1 in the main text.

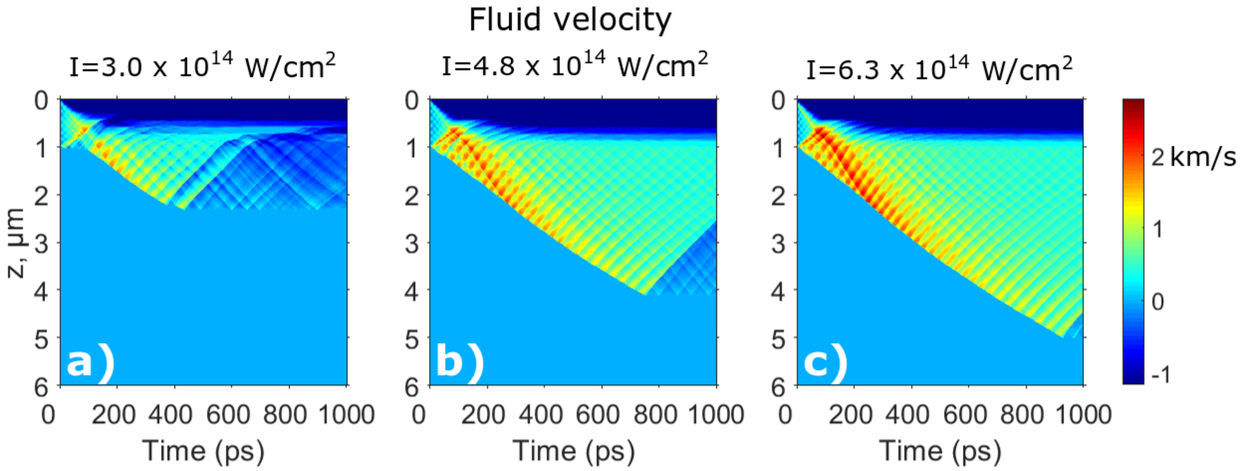

FIG. S11. Hydrodynamic simulations of the shock wave propagation. Color plots show simulation results for the fluid velocity (a-c) for three different IR laser intensities: (a) I_1=3.0 · 10^{14} W/cm^2, (b) I_2=4.8 · 10^{14} W/cm^2, (c) I_3=6.3 · 10^{14} W/cm^2.

C. Simulations using HELIOS code only

We performed another set of the hydrodynamic simulations for three IR laser intensity using only HELIOS program (Set 2). The simulation Set 2 was performed in order to compare PIConGPU coupled to HELIOS simulations and pure HELIOS simulations.

In the simulation Set 2 the experimental laser parameters were used. The simulations were performed for three experimental laser intensities (I_1=3.0 · 10^{14} W/cm^2, (b) I_2=4.8 · 10^{14} W/cm^2, (c) I_3=6.3 · 10^{14} W/cm^2) with 800 nm wavelength. The FWHM of the laser pulse was 50 fs and the peak laser power was set at 50 fs after the beginning of the simulation.

Percentage of power reflected at the critical surface was obtained from the PIConGPU simulations. The energy emitted by IR laser and the energy absorbed by electrons for three IR laser intensities is summarized in Table S2 and the absorption coefficient was around 10% for all three IR laser intensities. Therefore the reflection coefficient in the HELIOS

simulations was set to 90%. This value of the reflection coefficient is also confirmed by simple estimations. In case of dense plasma the absorption coefficient derived from the Fresnel formulas can be written in the form $A = 4\pi l_s/\lambda$, where A is the absorption coefficient, l_s is the skin depth and λ is the IR laser wavelength [13]. For the estimated skin depth of 10 nm the absorption coefficient is about 15 %.

In the simulation Set 2, the two-temperature model was used, similar to the previous case. In the two-temperature model both electrons and ions were assumed to have a room temperature in the initial state. All other parameters of the simulation, except the laser parameters were the same as in the previous case (simulation Set 1 summarized in the Methods section in the main text). The initial mass density of the colloidal crystal was similar to the previous simulation and had the same periodicity. The quiet start temperature was set to 0.044 eV which is equal to the polystyrene melting temperature.

The pressure and mass density obtained from these set of simulations are shown in Figs. S12,S13,S14. Two processes occur in the simulated colloidal crystal sample - ablation and shock propagation (the same processes were observed in simulation Set 1). Ablation is well seen in Fig. S12(d-e) as the zero mass density on the top of the sample. The top 1-2 layers are ablated during the first picoseconds and the ablation depth is summarized in Table S3. In the simulation Set 2 the ablation depth is slightly smaller than in the simulation Set 1 (see Fig. 5 in the main text and Fig. S10). Such a difference may be caused by the different IR laser absorption mechanisms implemented in these programs.

The maximum shock wave pressure achieved at the first picosecond of the simulation is on the order of 150-200 GPa (see Table S3 and Fig. S13(a-c)). At 20 ps the shock wavefront propagated through 0.5 μm of the colloidal crystal and destroyed the periodic structure of the sample. In the simulation Set 1 the periodicity of the first 1 μm was damaged 20 picoseconds after the beginning of the laser pulse. Therefore the simulation Set 2 is not explaining the fast drop of the diffracted intensity in the experimental results, while the simulation Set 1 the changes in the colloidal crystal structure can be attributed to the reduced diffracted intensity.

Further the shock wave is propagating through the colloidal crystal sample. The shock wave stops at the different time and depth summarized in Table S3. It is worth to notice that compare to simulation Set 1, here the shock wave stops earlier and destroys less of the material. The difference in the shock wave depth is on the order of 25% for two higher IR

laser intensities and approximately 1% for the lower IR laser intensity. Such a difference in the shock induced dynamics might be due to the different geometries (1D in HELIOS or 3D in PIConGPU). In the 3D PIConGPU simulations the colloidal crystal structure was properly set, while in the HELIOS simulation only a 1D projection of the mass density was used.

TABLE S2. The IR laser emitted and absorbed energy, and absorption coefficient estimated from PIConGPU simulations.

Intensity, 10^{14} W/cm^2	3.0	4.8	6.3
Laser emitted energy, 10^{10} eV	3.5	5.5	7.2
Absorbed energy, 10^9 eV	3.5	5.7	7.0
Absorption coefficient, %	10.0	9.6	9.7

TABLE S3. Results of the simulation Set 2 (performed only with HELIOS code on the periodic colloidal crystal sample). The results are shown for three IR laser intensities. Ablation depth and shock wave time and depth were calculated from the results of the hydrodynamic simulations.

Intensity, 10^{14} W/cm^2	3.0	4.8	6.3
Maximum shock wave pressure, GPa	143	184	214
Ablation depth, nm	150	180	300
Shock wave depth, μm	2.24	3.30	4.10
Simulated shock wave stop times, ps	377	589	751

D. HELIOS simulations for the bulk polystyrene sample

The last set of the hydrodynamics simulations was performed using only HELIOS code with the bulk polystyrene sample (Set 3). In this case all the simulations parameters were similar to the Set 2, except the initial polystyrene mass density. The polystyrene mass density was set to be constant along z - direction (1.05 g/cm^3) considering bulk polystyrene. The results of the simulation Set 3 are present in Fig. S15, S16, S17. The two main processes in the simulation Set 3 are ablation and the shock wave propagation, similar to simulation Set 1 and Set 2.

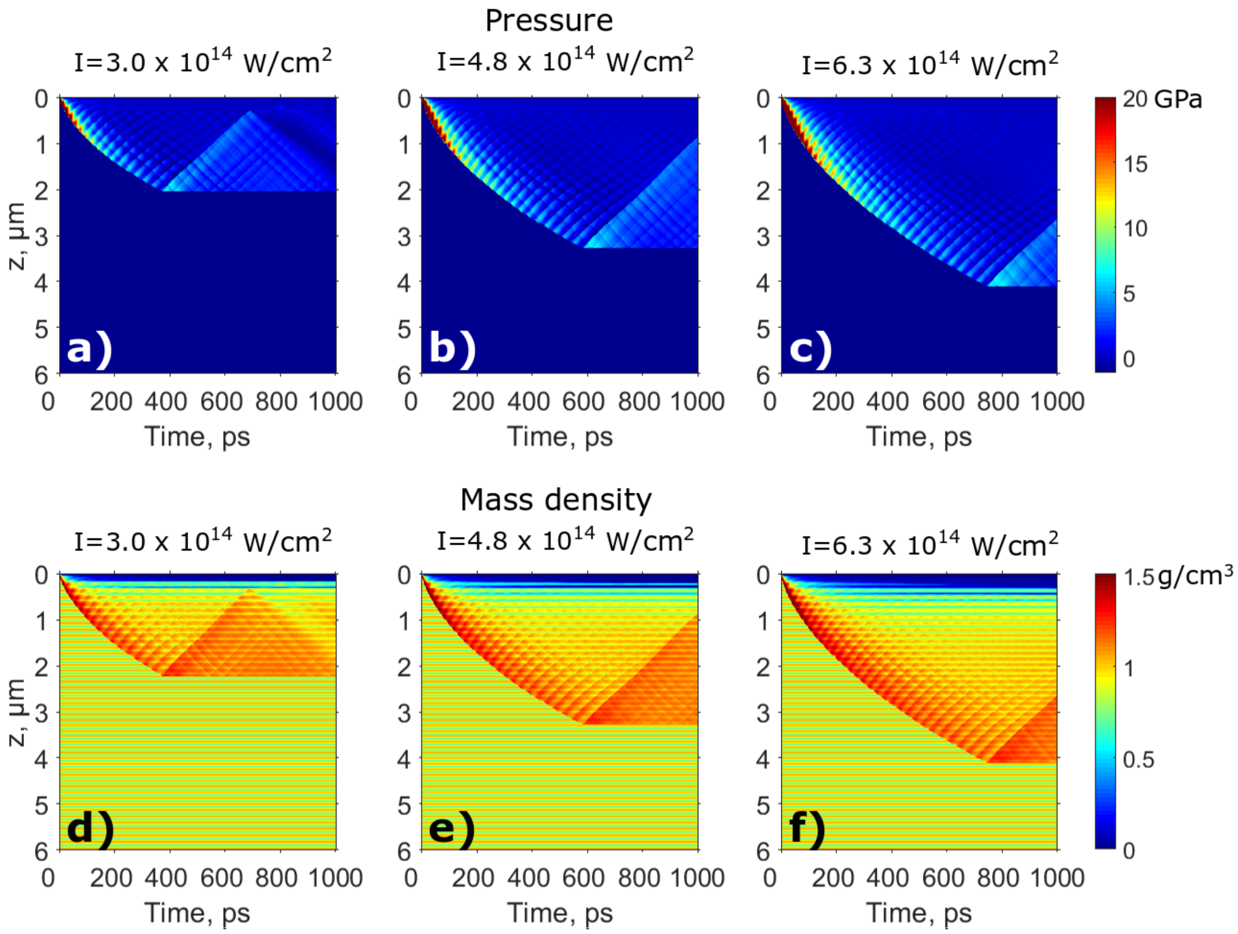

FIG. S12. Hydrodynamic simulations of the shock wave propagation inside the periodic colloidal crystal. The simulations were performed using only the HELIOS code. Color plots show simulation results for the pressure (a-c) and mass density (d-f) for three different IR laser intensities: (a,d) I_1=3.0 · 10^{14} W/cm^2, (b,e) I_2=4.8 · 10^{14} W/cm^2, (c-f) I_3=6.3 · 10^{14} W/cm^2.

The snapshot of the simulation at 1 ps is shown in Fig. S15(a-c) and S16(a-c), and the initial constant pressure and density of the sample is visible. The ablation of the material is already visible at 20 ps and it continues up to 100 ps (see Fig. S15, S17. The ablation depth is on the order of 100-200 nm and is summarized in Table. S4. In the simulation Set 3 the ablation depth was approximately twice smaller than for simulation Set 2. The difference in the ablation depth can be explained by smaller average mass density in case of the simulation Set 2.

The maximum pressure is reached at the first picosecond of the simulation on the very top layer of the polystyrene sample (see Fig. S16 and Table. S4). The pressure–depth dependence

had a periodic structure in the simulation Set 1 and Set 2 but in the simulation Set 3 it does not show any periodicity. Therefore we can conclude that such pressure modulations were caused by the periodic structure of the colloidal crystal sample.

The peak pressure is decaying while the shock wave is propagating inside the sample (see Fig. S16), but the shape of the shock wavefront remains stable. When the shock pressure is not sufficient to compress the material the shock wave stops and the shock wave stop time and depth are summarized in Table. S4. For the simulation Set 3 the shock wave stop time are much smaller than in case of simulation Set 2. The difference can be explained by the higher average mass density of the simulated sample.

TABLE S4. Results of the simulation Set 3 (performed only with HELIOS code on the periodic colloidal crystal sample). The results are shown for three IR laser intensities. Ablation depth and shock wave time and depth were calculated from the results of the hydrodynamic simulations.

Intensity, 10^{14} W/cm^2	3.0	4.8	6.3
Maximum shock wave pressure, GPa	148	196	225
Ablation depth, nm	80	120	180
Shock wave depth, μm	1.53	2.16	2.69
Simulated shock wave stop times, ps	301	454	581

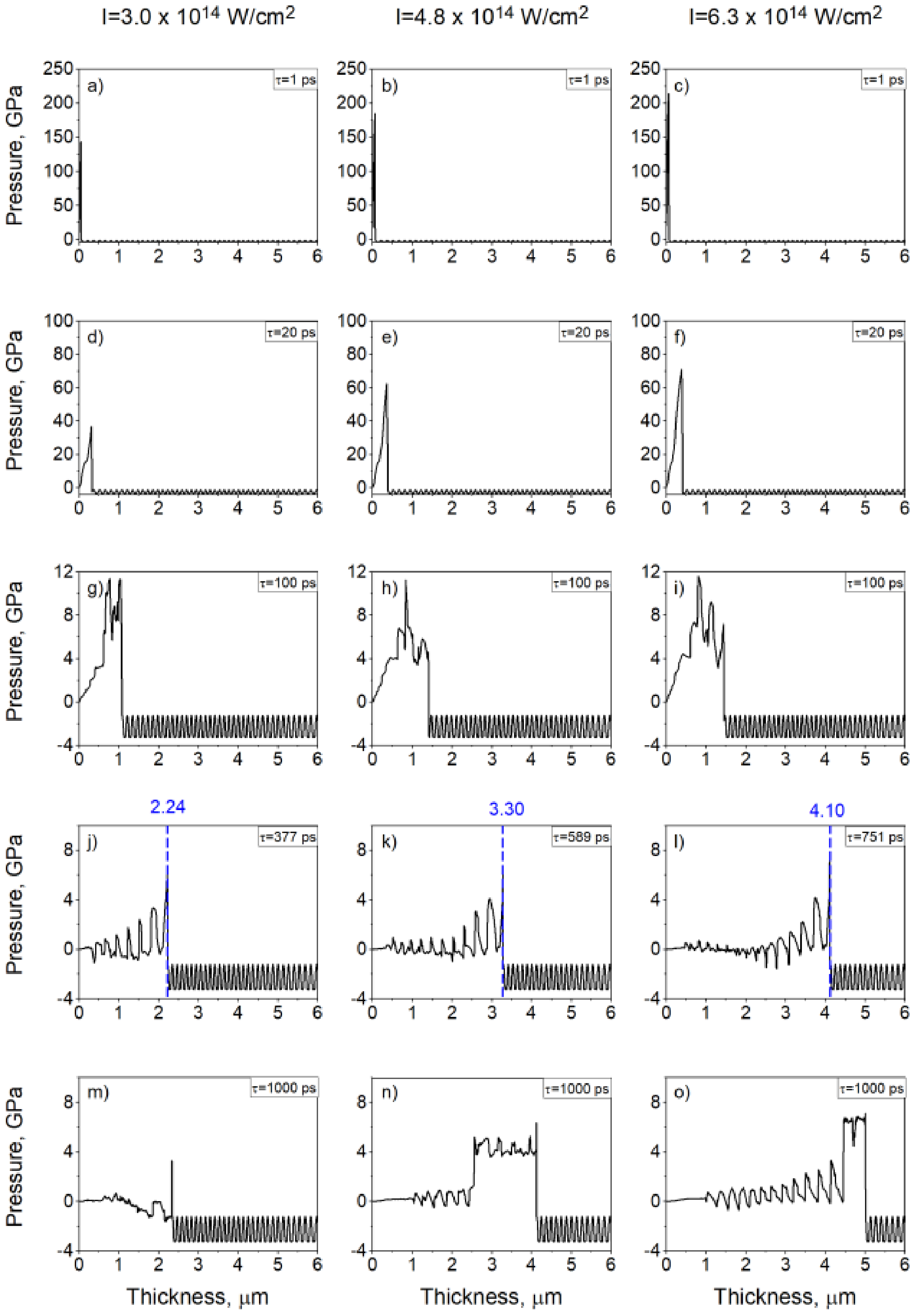

FIG. S13. Pressure in the colloidal crystal from the hydrodynamic simulation. The simulations were performed using only the HELIOS code. Results are shown at 1 ps (a-c), 20 ps (d-f), 100 ps (g-i) after the stop of the shock wave (j-l) and at 1000 ps, the end of the simulation (m-o). The blue dashed line shows the shock wave stop depth (j-l).

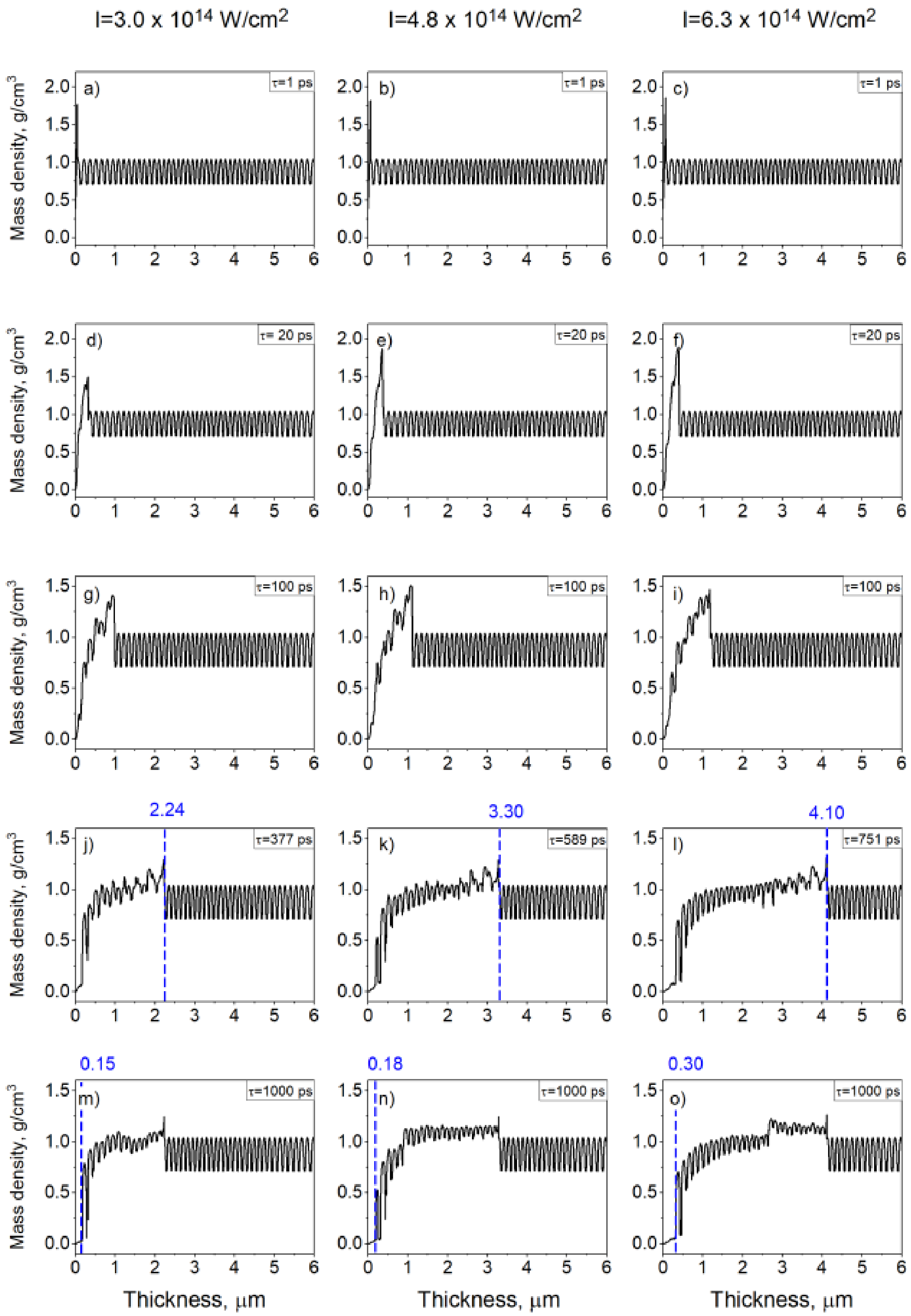

FIG. S14. Mass density of the periodic colloidal crystal from the hydrodynamic simulation. In this case only the HELIOS code was used. Results are shown at 1 ps (a-c), 20 ps (d-f), 100 ps (g-i) after the stop of the shock wave (j-l) and at 1000 ps, the end of the simulation (m-o). The blue dashed line shows the shock wave stop depth (j-l) and the ablation threshold (d-f).

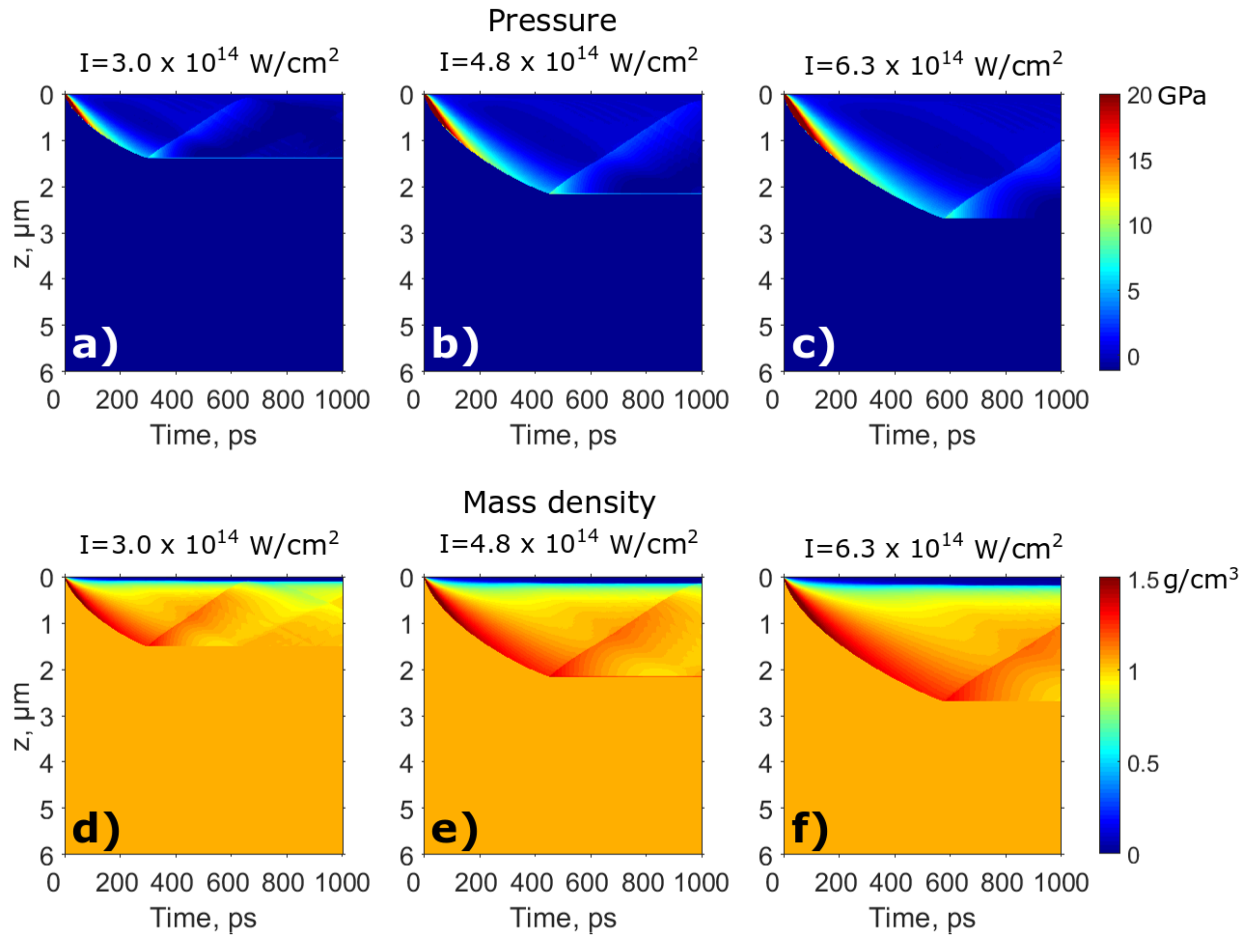

FIG. S15. Hydrodynamic simulations of the shock wave propagation inside the bulk polystyrene sample. In this case only the HELIOS code was used. Color plots show simulation results for the pressure (a-c) and mass density (d-f) for three different IR laser intensities: (a,d) I_1=$3.0 \cdot 10^{14}$ W/cm^2, (b,e) I_2=$4.8 \cdot 10^{14}$ W/cm^2, (c-f) I_3=$6.3 \cdot 10^{14}$ W/cm^2.

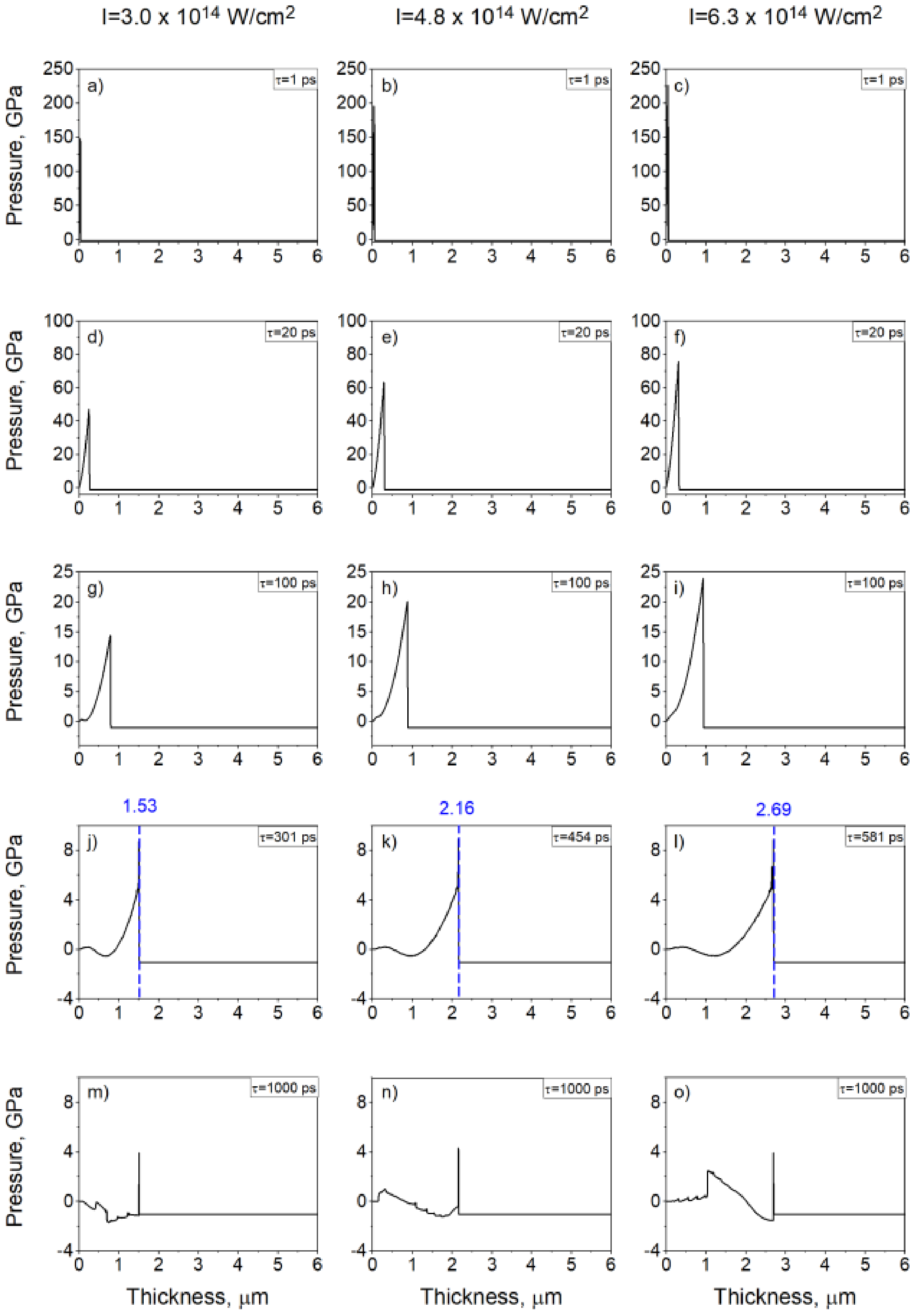

FIG. S16. Pressure inside the of the bulk polystyrene from the hydrodynamic simulation. In this case only the HELIOS code was used. Results are shown at 1 ps (a-c), 20 ps (d-f), 100 ps (g-i) after the stop of the shock wave (j-l) and at 1000 ps, the end of the simulation (m-o). The blue dashed line shows the shock wave stop depth (j-l).

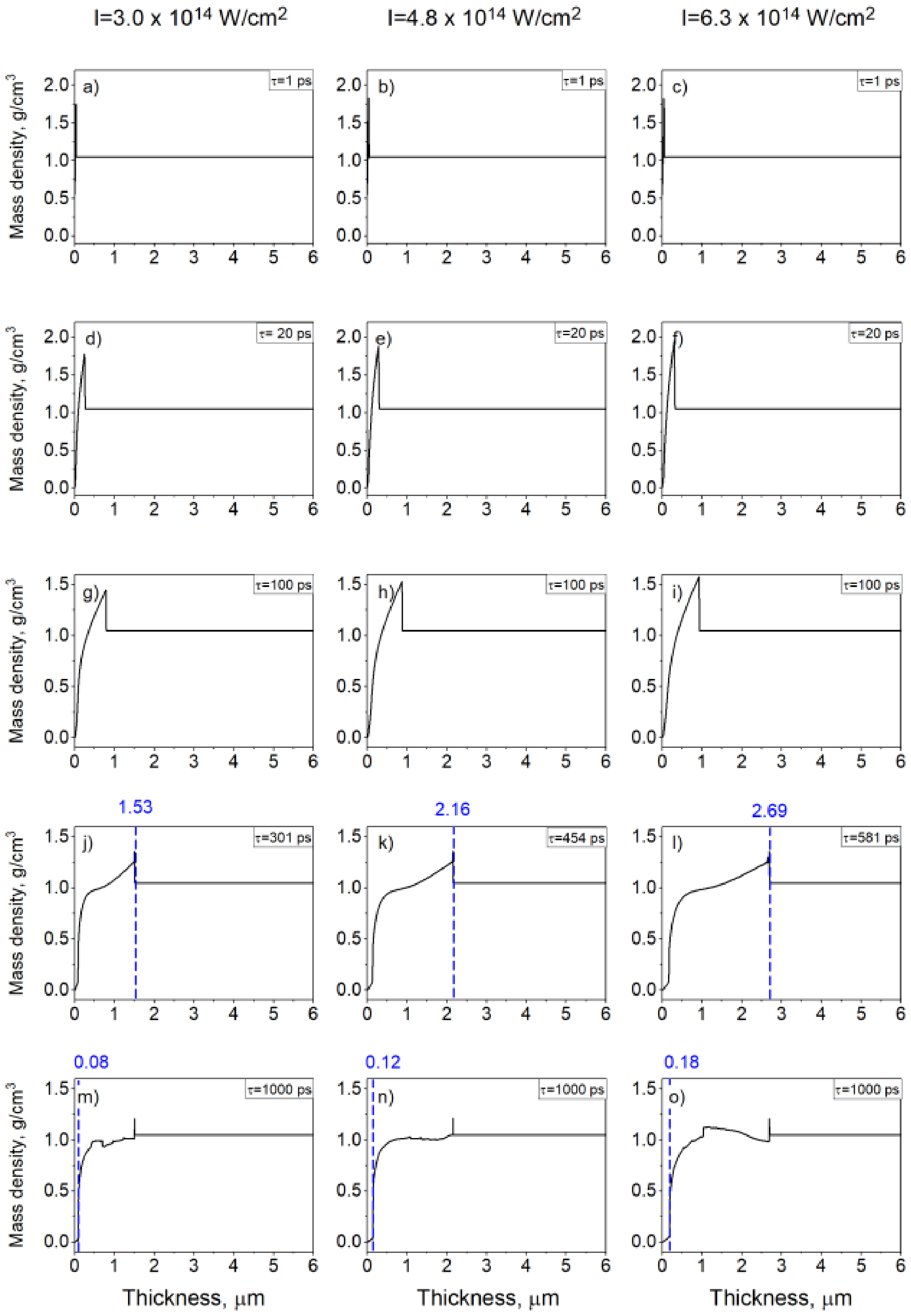

FIG. S17. Mass density of the bulk polystyrene from the hydrodynamic simulation. In this case only the HELIOS code was used. Results are shown at 1 ps (a-c), 20 ps (d-f), 100 ps (g-i) after the stop of the shock wave (j-l) and at 1000 ps, the end of the simulation (m-o). The blue dashed line shows the shock wave stop depth (j-l) and the ablation threshold (d-f).

[1] Mukharamova, N. *et al.* Probing dynamics in colloidal crystals with pump-probe experiments at LCLS: Methodology and analysis. *Appl. Sci.* **7**, 519 (2017).

[2] Burau, H. *et al.* PIConGPU: A fully relativistic particle-in-cell code for a GPU cluster. *IEEE Transactions on Plasma Science* **38**, 2831–2839 (2010).

[3] Bussmann, M. *et al.* Radiative signatures of the relativistic kelvin-helmholtz instability. In *Proceedings of the International Conference on High Performance Computing, Networking, Storage and Analysis*, SC '13, 5:1–5:12 (2013).

[4] Courant, R., Friedrichs, K. & Lewy, H. On the partial difference equations op mathematical physics. *Mathematische Annalen* **100**, 32–74 (1928).

[5] Yee, K. Numerical solution of initial boundary value problems involving maxwell's equations in isotropic media. *IEEE Transactions on antennas and propagation* **14**, 302–307 (1966).

[6] Huebl, A. *et al.* openPMD 1.0.0: A meta data standard for particle and mesh based data. (2015). (Acknowledgements) The design of this standard is based on the experiences we gained in the last years implementing efficient, parallel I/O in several PIC codes, but most recently in PIConGPU (picongpu.hzdr.de). Special thanks goes to Felix Schmitt (formerly ZIH / TU Dresden, now with Nvidia Corp.) and Ren Widera (HZDR).

[7] Keldysh, L. Ionization in the field of a strong electromagnetic wave. *Sov. Phys. JETP* **20**, 1307–1314 (1965).

[8] Reiss, H. The tunnelling model of laser-induced ionization and its failure at low frequencies. *J. Phys. B: At. Mol. Opt. Phys.* **47**, 204006 (2014).

[9] Reiss, H. Limits on tunneling theories of strong-field ionization. *Phys. Rev. Lett.* **101**, 043002 (2008).

[10] Krainov, V. P. & Smirnov, M. B. Cluster beams in the super-intense femtosecond laser pulse. *Phys. Rep.* **370**, 237–331 (2002).

[11] Delone, N. B. & Krainov, V. P. Tunneling and barrier-suppression ionization of atoms and ions in a laser radiation field. *Physics-Uspekhi* **41**, 469–485 (1998).

[12] More, R. Pressure ionization, resonances, and the continuity of bound and free states. *Advances in atomic and molecular physics* **21**, 305–356 (1985).

[13] Gamaly, E. G., Rode, A. V., Luther-Davies, B. & Tikhonchuk, V. T. Ablation of solids by

femtosecond lasers: Ablation mechanism and ablation thresholds for metals and dielectrics. *Physics of plasmas* **9**, 949–957 (2002).
